# Prefrontal Cortex 5‐HT1A Receptor‐Coupled Inwardly Rectifying Potassium Channels Decreased Seizure Susceptibility in Rat Models With Autism Spectrum Disorder

**DOI:** 10.1155/np/4005949

**Published:** 2026-05-05

**Authors:** Zhuoqi Li, Yangyang Sun, Xianhao Huo, Xiaofan Ren, Na Ding, Xin Qian, Ao Li, Tao Sun

**Affiliations:** ^1^ Department of Neurosurgery, General Hospital of Ningxia Medical University, Yinchuan, Ningxia, China, nxmu.edu.cn; ^2^ Ningxia Key Laboratory of Craniocerebral Diseases, Ningxia Medical University, Yinchuan, Ningxia, China, nxmu.edu.cn; ^3^ School of Clinical Medicine, Ningxia Medical University, Yinchuan, Ningxia, China, nxmu.edu.cn

**Keywords:** 5-HT1A receptor, autism spectrum disorder, excitatory/inhibitory imbalance, inwardly rectifying potassium channel, prefrontal cortex, seizure susceptibility

## Abstract

Dysregulation of serotonin 1A receptor (5‐HT1A), a G protein‐coupled inhibitory receptor, is implicated in the pathogenesis of both autism spectrum disorder (ASD) and epilepsy. The prefrontal cortex (PFC) is particularly vulnerable to the factors that affect neuronal and synaptic development, with abnormal PFC development leading to increased epilepsy susceptibility. This study used 8‐OH‐DPAT to activate PFC 5‐HT1A to investigate its role in attenuating epileptic susceptibility in a valproic acid (VPA)‐induced rat model of ASD and potential mechanisms involving Kir3 channel‐mediated hyperpolarization. Rats were prenatally exposed to VPA to induce autism‐like behaviors, and successful induction was verified through behavioral, morphological, and electrophysiological assessments. Neuronal loss, dendritic complexity, and spine density in the PFC were evaluated using Nissl and Golgi staining. Pentylenetetrazol (PTZ) was used to induce chemical kindling for assessing seizure susceptibility in the ASD model. Spontaneous action potential (sAP) and miniature excitatory postsynaptic current (mEPSC) frequencies were electrophysiologically recorded. The selective 5‐HT1A receptor (5‐HT1AR) agonist 8‐OH‐DPAT was used to investigate its anticonvulsant effects. ASD rats exhibited significant neuronal loss, reduced dendritic complexity, and lower dendritic spine density in the PFC. The PTZ‐treated ASD group showed reduced seizure onset latency, prolonged stage IV seizure duration, and higher seizure incidence, indicating increased susceptibility to epilepsy. Untreated rats displayed reduced sAP and mEPSC frequencies in PFC pyramidal neurons, suggesting E/I imbalance. However, PTZ treatment increased sAP and mEPSC frequencies, reflecting enhanced neuronal excitability. Treatment with 8‐OH‐DPAT significantly delayed seizure onset, shortened seizure duration, and reduced seizure incidence. Furthermore, 8‐OH‐DPAT decreased sAP and mEPSC frequencies. These effects were attenuated after applying tertiapin‐Q (TQ), underscoring the role of inwardly rectifying potassium (Kir3) channels in mediating 8‐OH‐DPAT‐induced anticonvulsant effects. In conclusion, PFC 5‐HT1AR activity alleviated epileptic activity through Kir3 channel‐mediated hyperpolarization. These findings highlight 5‐HT1ARs and Kir3 channels as promising therapeutic targets for epilepsy associated with ASD.

## 1. Introduction

Autism spectrum disorder (ASD), a condition of neurodevelopmental origin, is characterized by impairments or delays in social and communication skills and repetitive behavioral patterns [1]. ASD often co‐occurs with conditions such as epilepsy, sleep disorders, anxiety, immune dysfunction, and gastrointestinal issues [2–6]. Among these, epilepsy—defined by recurrent seizures affecting the central nervous system—is the most common comorbidity in patients with ASD [7]. Compared with people without ASD, individuals with ASD have higher rates of epilepsy (21% vs. 0.8%) [8]. Given the association of epilepsy with developmental delays or intellectual disabilities [9], investigating the pathological mechanisms shared by ASD and epilepsy is essential.

Neurobiological studies on ASD have focused on two main areas—brain morphology and structural abnormalities. Many individuals with ASD exhibit small head circumferences at birth [10] but experience rapid and abnormal growth, particularly in the frontal cortex, cerebellum, and limbic system, among other brain regions, by ages of 2–4 years. Structural abnormalities associated with ASD include reduced neuronal and dendritic complexity, mainly in the frontal cortex, hippocampus, and amygdala [11]. The prefrontal cortex (PFC) is a key region for cognition, emotion, and social function [12], and impairments in PFC development are often associated with ASD [13]. Most studies have relied on ASD animal models, generated through prenatal exposure to valproic acid (VPA), which induces significant local hyperconnectivity in neocortical pyramidal neurons and reduces intrinsic excitability and putative synaptic connections between layer 5 pyramidal neurons [14]. Local hyperconnectivity may enhance sensitivity to external stimuli [15], as substantiated by a study in VPA‐exposed *Xenopus laevis* tadpoles that displayed increased susceptibility to pentylenetetrazol (PTZ)‐induced seizures [16], correlating with neural network hyperconnectivity, increased synaptic (both excitatory and inhibitory) drive, and excessive spontaneous synaptic activity.

ASD research primarily centers around the serotonergic (5‐HT) systems, which are also considered a biomarker for ASD and other psychiatric disorders [17]. The 5‐HT1A receptor (5‐HT1AR), a G protein‐coupled receptor, is an inhibitory neurotransmitter receptor that functions at both presynaptic and postsynaptic regions and mediates neuronal activity changes upon binding with serotonin (5‐HT) [18]. A study in ASD rats revealed that 5‐HT, its metabolites, and 5‐HT1AR exhibited altered expression in the anterior insular cortex, suggesting that 5‐HT1AR‐mediated cortical excitability is impaired in ASD. The 5‐HT systems are also involved in epilepsy [19]. The hippocampal density of 5‐HT1AR was found to be low in rats with a genetic predisposition to epilepsy [20]. Similarly, individuals with temporal lobe epilepsy demonstrated reduced 5‐HT1AR binding potential in the anterior cingulate cortex, temporal cortex, and insular cortex [21–23]. In kindling epilepsy models, 5‐HT1AR antagonists were reported to promote seizures [24], whereas 5‐HT1AR agonists reduced seizure frequency, potentially through neuronal hyperpolarization [25]. The 5‐HT1AR couples with multiple G proteins, including G_αi/o_, and is linked to inwardly rectifying potassium (Kir3) channels, which, upon activation, induce hyperpolarization in pyramidal neurons [26]. The PFC, densely innervated by serotonergic axons, is enriched in various 5‐HTR subtypes, particularly 5‐HT1A and 5‐HT2A [27]. Taken together, the results of the above literature reveal that the role 5‐HT1ARs in the PFC in the study of epilepsy susceptibility in autistic rats and their specific mechanism of action have not yet been reported.

The present study investigated the susceptibility to epilepsy in ASD rat models. In addition, it examined the role of PFC 5‐HT1AR activity in modulating this susceptibility and the potential mechanism underlying its effect, focusing on 5‐HT1AR‐coupled Kir3 channels.

## 2. Materials and Methods

### 2.1. Animals

Sprague‐Dawley rats, weighing 230–250 g, were obtained from the Ningxia Medical University Animal Center, China. The rats were housed under a controlled environment with a 22 ± 2°C temperature, a 12 h light/dark cycle, and ad libitum access to food and water. Each cage contained 4–5 rats. Behavioral tests were conducted between 9:00 and 17:00. All animal experiments and care protocols were in accordance with the Guide for the Care and Use of Laboratory Animals published by the National Institutes of Health (NIH) and approved by the Institutional Animal Care and Use Committee (IACUC) of Ningxia Medical University (Approval Number: IACUC‐NYLAC‐2024‐010). Experiments were meticulously designed to ensure minimal harm to the animals and minimize the number of animals required.

### 2.2. VPA‐Induced ASD Model

To avoid maternal mortality or potential miscarriage, a previously approved rat model [28] was referenced and improved upon in this study. Sodium valproate (P4543, Sigma‐Aldrich, USA) was dissolved in 0.9% saline to prepare a 200 mg/mL solution (pH = 7.3). Female Sprague‐Dawley rats carrying fetuses were administered a single intraperitoneal injection of valproate (500 mg/kg) on embryonic day 12.5, whereas control rats received an equal dose of 0.9% saline. Offspring were weaned on postnatal day 21 and segregated by sex; 4–5 rats were kept in each cage. Male offspring exposed to VPA and saline at 4–6 weeks of age were used as experimental and control rats, respectively, for subsequent experiments.

### 2.3. Nissl Staining

The rats were anesthetized using isoflurane and perfused transcardially with saline, followed by fixation with 4% paraformaldehyde. The whole brain was extracted and fixed in a general‐purpose tissue fixative for 24 h. The samples were dehydrated in an ethanol gradient and then subjected to xylene clearance and paraffin embedding. Brain tissues containing the PFC were coronally sectioned (5 µm), and the sections were deparaffinized, rehydrated, and stained with Nissl stain (G1432, Solarbio, China) for 15 min. Subsequently, the sections were dehydrated in ethanol, cleared with xylene, and mounted with neutral resin. Morphological changes in PFC neurons were observed and imaged under a light microscope (BX53, Olympus, Japan). For neuronal quantification, images of Nissl‐stained sections were acquired using ZYEviewer software, and cell counting was performed in a single field of view at 40× magnification. Neurons displaying preserved morphology, intact ring‐like Nissl bodies, and clearly visible nuclei were defined as positive cells. Quantification was performed using ImageJ software. To ensure objectivity, all analyses were performed by an investigator blinded to group allocation, with sections identified only by coded numbers. Data are presented as the mean value per animal (*n* = 3 rats per group), and each value represents the average obtained from six fields of view.

### 2.4. Golgi Staining

Golgi staining was performed using the FD Rapid Golgi Stain Kit (PK401, FD NeuroTechnologies, USA). First, the rats were anesthetized with isoflurane and decapitated. Whole brains were immersed in the impregnation solution (10 mL Solution A + 10 mL Solution B) at room temperature for 24 h, after which the solution was replaced. The samples were stored in the dark for 24 days, then transferred to Solution C (15 mL) and incubated for 7 days, and the solution was replaced after 24 h. Coronal brain slices (100 μm) were prepared using a vibratome (VT1000S, Leica, Germany) and incubated in a staining solution (5 mL Solution D + 5 mL Solution E + 10 mL distilled water) for 10 min, followed by dehydration with an ethanol gradient and clearance with xylene. Imaging of PFC dendrites was performed at 20× magnification, and dendritic spines were imaged using a 100× oil‐immersion objective with a compound light microscope (DM2000, Leica Microsystems, Germany) equipped with a digital camera (DFC450C, Leica Microsystems, Germany). Dendritic spine quantification was performed using the NeuroJ plugin (v1.1.0) in ImageJ software (v1.54d, National Institutes of Health, Bethesda, MD, USA). For each neuron, three 50 μm dendritic segments were selected from third‐order apical dendritic branches located 50–150 μm from the soma to ensure uniform sampling across cells. Based on established morphological criteria, spines were classified as thin, mushroom, or stubby. Thin spines were defined by a long neck and small head, mushroom spines by a prominent head with a constricted neck, and stubby spines by a short protrusion without an obvious neck. Three‐dimensional (3D) reconstruction of dendritic architecture was performed using Neurolucida 360 software (v2022.1, MBF Bioscience, Williston, VT, USA), and dendritic branching patterns and structural complexity were analyzed using Neurolucida Explorer software (v1.12.0). Sholl analysis was performed by quantifying dendritic intersections at concentric circles drawn at 10 μm intervals from the cell soma, and neuronal complexity was further evaluated by measuring dendritic intersections and total dendritic length at a radial distance of 200 μm from the cell body. For sampling, three rats were included in each experimental group, and six neurons per rat were randomly selected from the anterior, middle, and posterior regions of the PFC, with two neurons analyzed from each region to ensure representative sampling. Only neurons exhibiting complete impregnation, uniform staining, minimal background interference, and clearly discernible dendrites and spines were included. All analyses were performed by an experimenter blinded to group assignment, and statistical comparisons were conducted using the appropriate parametric or nonparametric tests as indicated.

### 2.5. Real‐Time Quantitative Polymerase Chain Reaction (RT‐qPCR)

The rats were deeply anesthetized with isoflurane (6% induction) and decapitated to isolate the PFC. The obtained samples were flash‐frozen in liquid nitrogen and stored at −80°C. Total RNA was extracted using the TRIzol reagent (15596018, Thermo Fisher Scientific, USA) and reverse‐transcribed into cDNA by using the TRUEscript First‐Strand cDNA Synthesis Kit (PC1803, Aidlab Biotechnologies, China). RT‐qPCR was performed using the SsoAdvanced Universal SYBR Green Supermix (1725275, Bio‐Rad, USA) and the 7300 Real‐Time PCR System (Applied Biosystems, Thermo Fisher Scientific, USA). The threshold cycle (CT) reading was collected. The relative expression of the target gene mRNAs was normalized to that of glyceraldehyde 3‐phosphate dehydrogenase (GAPDH; B661204, Sangon Biotech, China) in all samples and calculated using the 2^−ΔΔCT^ method. Primer sequences used are as follows:

5‐HT1AR:

Forward: 5′‐CGTGCACCATCAGCAAGGA‐3′.

Reverse: 5′‐CTGAAGATGCGCCCGTAGAGA‐3′.

### 2.6. Western Blot

Sample preparation was performed as described above. Tissues were homogenized using a Vibra‐Cell ultrasonic homogenizer (#VCX 130PB; Sonics & Materials Inc., Newtown, CT, USA) in lysis buffer containing phosphatase inhibitors, protease inhibitors, and phenylmethylsulfonyl fluoride (PMSF). Following centrifugation at 12,500 × g for 10 min at 4°C, the supernatants were collected for protein quantification. Protein extracts were mixed with loading buffer and denatured at 100°C for 10 min prior to electrophoresis. Equal amounts of protein were separated by 10% SDS‐PAGE and transferred onto 0.45 μm polyvinylidene difluoride membranes (Millipore, Billerica, MA, USA). The membranes were blocked with Rapid Blocking Buffer (ABclonal, Wuhan, China) for 10 min at 28–30°C, followed by overnight incubation at 4°C with rabbit anti‐5‐HT1AR antibody (1:1000, Abcam, ab227165) and anti‐GAPDH antibody (1:5000, Affinity, AF7021). After washing three times with TBST, the membranes were incubated for 1 h with IRDye800CW Goat anti‐Rabbit IgG secondary antibody (1:1000, LI‐COR Biosciences, 926‐32211). Following additional TBST washes, protein bands were detected using the Odyssey CLX imaging system (LI‐COR, USA), and densitometric analysis was performed using ImageJ software. The uncropped original images of the WB experiment results are presented in Supporting Information 1: Figure S1 and Supporting Information 2: Figure S2.

### 2.7. Electroencephalographic (EEG) Recordings and Cannula Implantation

The rats were anesthetized with isoflurane and secured in a stereotaxic apparatus. After exposing the skull, recording electrodes were implanted at 3 mm posterior to the bregma [29] and 2 mm lateral to the midline [30]. Reference electrodes were placed in the cerebellar region. Guide cannulas were implanted in the PFC (2.5–4.5 mm anterior to the bregma, 0.1–1.1 mm lateral, 2.5–3.0 mm depth) and secured with dental cement. Following a 7‐day recovery period, the rats were administered an injection of a 5‐HT1AR agonist, 8‐hydroxy‐2‐(di‐n‐propylamino)tetralin (8‐OH‐DPAT; HY‐112061, MCE, China; 0.3 mg/kg); 20 min after 8‐OH‐DPAT injection, the EEG measurements were performed. Baseline EEG was recorded for 5 min, followed by the induction of seizures through the administration of PTZ (P6500, Sigma‐Aldrich, USA; 60 mg/kg). EEG changes were recorded for 4 h after PTZ administration and analyzed using BL‐420 software.

### 2.8. Preparation of Brain Slices for Whole‐Cell Patch‐Clamp Recording

Control and ASD model rats (4–6 weeks old) were euthanized and decapitated. Whole brains were extracted to prepare slices, which were placed immediately in ice‐cold artificial cerebrospinal fluid (ACSF) continuously oxygenated with 95% O_2_/5% CO_2_; ACSF comprised 124 mM NaCl, 24 mM NaHCO_3_, 3 mM KCl, 1.25 mM NaH_2_PO_4_, 2 mM CaCl_2_, 1.5 mM MgCl_2_, and 10 mM glucose. The ice‐cooled samples were trimmed and sliced into 350 μm‐thick sections by using a vibratome. The slices were then transferred to oxygenated ACSF at room temperature and incubated for 1 h before whole‐cell patch‐clamp recordings. The slices were perfused with flowing ACSF at a rate of 4 mL/min for recordings. Layer V pyramidal neurons in the PFC were selected for the experiments. Three rats per group and three cells per rat were selected for recording.

#### 2.8.1. Spontaneous Action Potential (sAP) Recording

Glass pipettes were filled with an intracellular solution containing 130 mM potassium gluconate, 10 mM HEPES, 2 mM MgCl_2_, 0.1 mM CaCl_2_, 2.54 mM ATP‐Na_2_, and 0.3% biocytin. The neurons’ membrane potential was recorded in the absence of any external stimulation to assess sAP in terms of frequency and amplitude.

#### 2.8.2. Miniature Excitatory Postsynaptic Current (mEPSC) Recording

Glass pipettes were filled with an intracellular solution containing 10 mM CsCl, 5 mM NMG, 4 mM NaCl, 1 mM MgCl_2_, 1 mM EGTA, 130 mM CsMeS, 1 mM HEPES, 12 mM phosphocreatine, 5 mM MgATP, 0.5 mM Na_2_GTP, and 0.3% biocytin (pH, 7.2; osmolarity, 290–310 mOsm). The extracellular solution was supplemented with 1 μM tetrodotoxin (TTX) and 50 μM picrotoxin (PTX). The mEPSCs were recorded at a holding potential of −70 mV.

#### 2.8.3. Miniature Inhibitory Postsynaptic Current (mIPSC) Recording

Pipettes were filled with an intracellular solution containing 62.5 mM potassium D‐gluconate, 8 mM NaCl, 62.5 mM KCl, 0.2 mM EGTA, 10 mM HEPES, 0.3 mM Na_2_GTP, 2 mM MgATP, and 0.3% biocytin (pH, 7.2; osmolarity, 290–310 mOsm). The extracellular solution contained 1 μM TTX, 50 μM DL‐AP5, 20 μM CNQX, and 100 μM GABA. The mIPSCs were recorded at a holding potential of −70 mV.

#### 2.8.4. Potassium Current Recording

Pipettes were filled with an intracellular solution containing 122.5 mM potassium D‐gluconate, 8 mM KCl, 4 mM MgATP, 10 mM phosphocreatine, 0.3 mM Na_2_GTP, 10 mM HEPES, 0.2 mM EGTA, 4 mM MgCl_2_, and 0.3% biocytin (pH, 7.2; osmolarity, 290–310 mOsm). The extracellular solution was supplemented with 10 μM DNQX, 50 μM DL‐AP5, 1 μM gabazine, 10 μM LY‐367385, and 1 μM CGP‐55845. Potassium currents were recorded at a holding potential of −70 mV, with step protocols ranging from −110 to −30 mV in 10 mV increments to measure corresponding currents. I–V curves were plotted according to the stimulus voltage and the resulting current response.

### 2.9. Statistical Analysis

Statistical analyses were performed using the independent‐samples *t*‐test and one‐way or two‐way ANOVA in GraphPad Prism software (Version 9.50). In case of unequal variance, rank tests were performed. Data are presented as the mean ± standard error of the mean. A *p* value of <0.05 was considered statistically significant.

## 3. Results

### 3.1. PFC Neurons Displayed Abnormal Development in ASD Rat Models

We determined the morphological features of PFC neurons as well as neuronal and dendritic spine density through Nissl and Golgi staining in the ASD model and control groups. PFC neurons in the control group exhibited intact morphology and clear outlines, whereas those in the ASD model group displayed atrophy and a looser arrangement (Figure 1). The number of positive cells was significantly higher in the control group than in the ASD model group (57.56 ± 12.92 vs. 24.26 ± 5.33, *p* = 0.0145). Golgi staining (Figure 2) showed that the density of dendritic spines in pyramidal neurons of the PFC was significantly reduced in the ASD model group compared with the control group (0.76 ± 0.02 vs. 1.04 ± 0.02, *p* < 0.01). Specifically, the densities of both thin and mushroom spines were decreased, while no significant change was observed in stubby spines (Figure 2C, D). Furthermore, we found that the number of dendritic intersections, length, and dendritic area (Figure 2I–K) of PFC neurons were all decreased in the ASD model group. The ASD group exhibited a decrease in dendritic branching in PFC pyramidal neurons, indicating that the prenatal exposure to VPA significantly impacted dendritic complexity, as revealed by Sholl analysis. Overall, the decrease in neuronal complexity and dendritic spine density in the PFC of ASD rats highlighted that the structural deficits in the brain caused alterations in the cortical function in the ASD rat model.

Figure 1Neuronal loss and decreased 5‐HT1A receptor expression in the prefrontal cortex of ASD model rats. (A–B) Representative Nissl‐stained coronal sections of the prefrontal cortex (PFC) from control rats (A) and ASD model rats (B). Compared with the control group, the ASD model group exhibited a reduced number of neurons and disrupted cellular organization in the PFC. (C) Quantification of positive cells in the PFC. The number of positive cells was significantly lower in the ASD model group than in the control group. Data are presented as mean ± SEM (*n* = 3 per group). Statistical significance was assessed using an independent‐samples *t*‐test.  ^∗^ *p* < 0.05. (D) Relative mRNA expression of the 5‐HT1A receptor in the PFC. The ASD model group showed significantly reduced 5‐HT1A receptor mRNA expression compared with the control group. Data are presented as mean ± SEM (*n* = 3 per group). Statistical significance was assessed using an independent‐samples *t*‐test.  ^∗^ *p* < 0.05. (E) Representative Western blot images showing 5‐HT1A receptor (56 kDa) and GAPDH (37 kDa) protein expression in the PFC of control and ASD model rats. (F) Quantitative analysis of 5‐HT1A receptor protein expression normalized to GAPDH. The ASD model group showed significantly lower 5‐HT1A receptor protein levels than the control group. Data are presented as mean ± SEM (*n* = 3 per group). Statistical significance was assessed using an independent‐samples *t*‐test.  ^∗^ *p* < 0.05).

**Figure:**
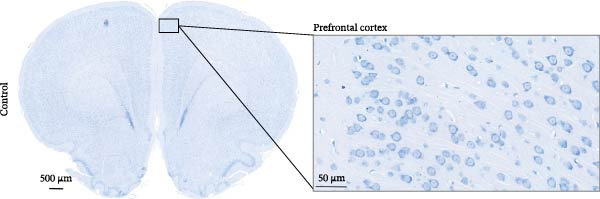
(A)

**Figure:**
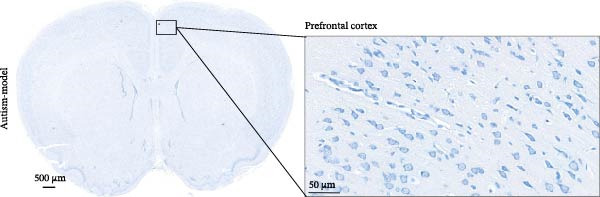
(B)

**Figure:**
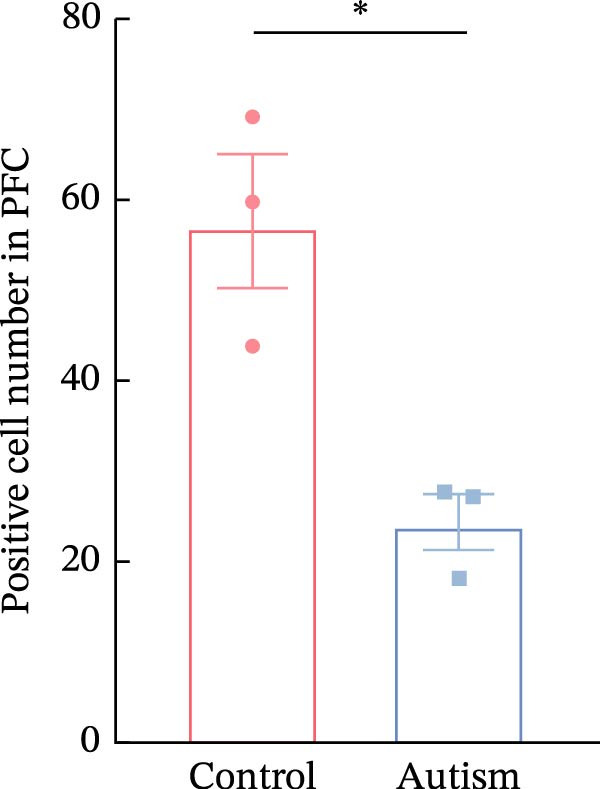
(C)

**Figure:**
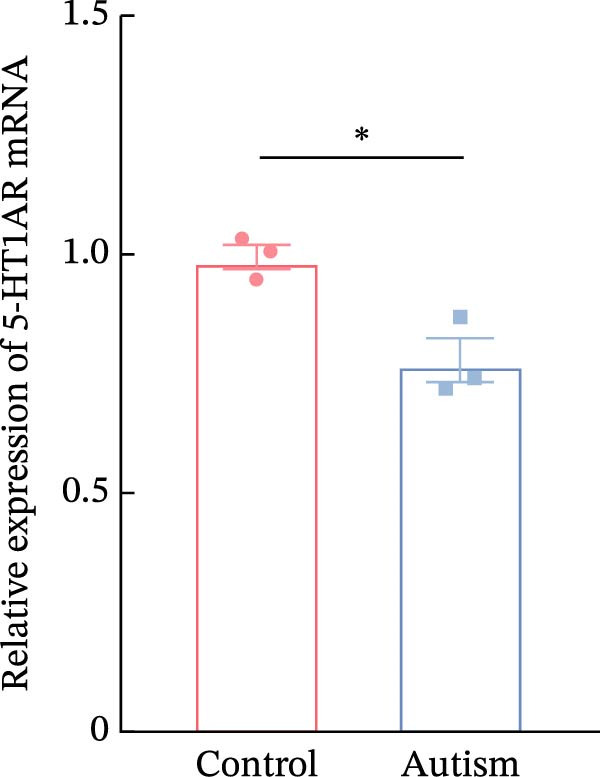
(D)

**Figure:**
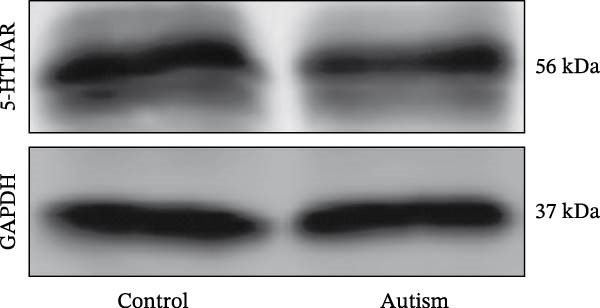
(E)

**Figure:**
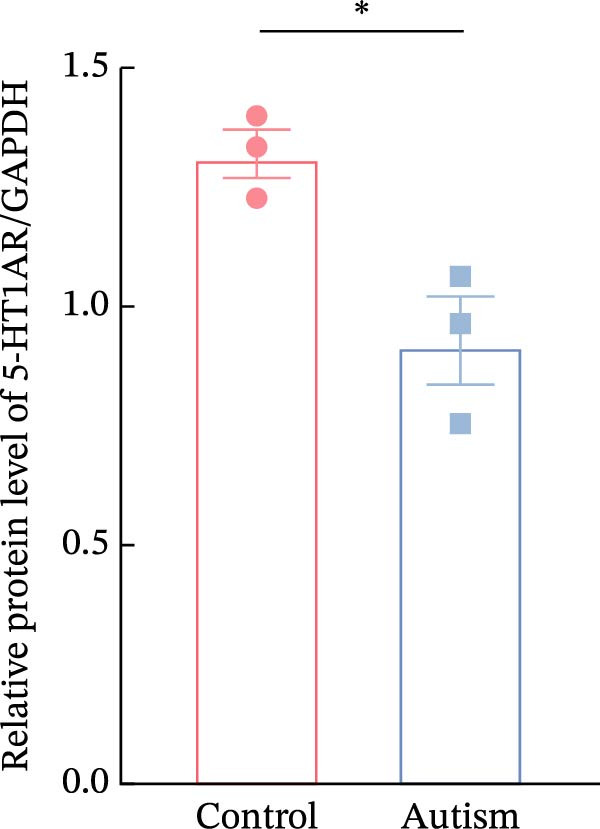
(F)

Figure 2Prefrontal cortex pyramidal neurons of ASD rats had reduced dendritic complexity and spine density. (A) Representative Golgi‐stained dendritic spines from pyramidal neurons in the prefrontal cortex (PFC) of control and ASD model rats; spine density was notably reduced in the ASD model group. (B) Quantification of total dendritic spine density; the number of dendritic spines was significantly lower in the ASD model group than in controls. (C) Quantification of thin dendritic spine density. The density of thin spines was significantly lower in the ASD model group than in the control group. (D) Quantification of mushroom dendritic spine density. Mushroom spine density was also significantly decreased in the ASD model group compared with controls. (E, F) Sholl analysis of PFC pyramidal neurons in the control (E) and ASD model groups (F), demonstrating reduced dendritic branching complexity in the ASD model group. (G, H) Representative three‐dimensional (3D) reconstructions of pyramidal neurons from Golgi‐stained PFC sections in the control (G) and ASD model groups (H), highlighting structural differences. (I–K) Sholl analysis plots showing the number of dendritic intersections (I), total dendritic length (J), and dendritic surface area (K) as a function of distance from the soma. All parameters were significantly reduced in the ASD model group compared with controls. Data are presented as mean ± SEM (*n* = 3 per group). Statistical significance was determined using two‐way ANOVA.  ^∗^ *p* < 0.05,  ^∗∗^ *p* < 0.01,  ^∗∗∗^ *p* < 0.001,  ^∗∗∗∗^ *p* < 0.0001, ns: not significant.

**Figure:**
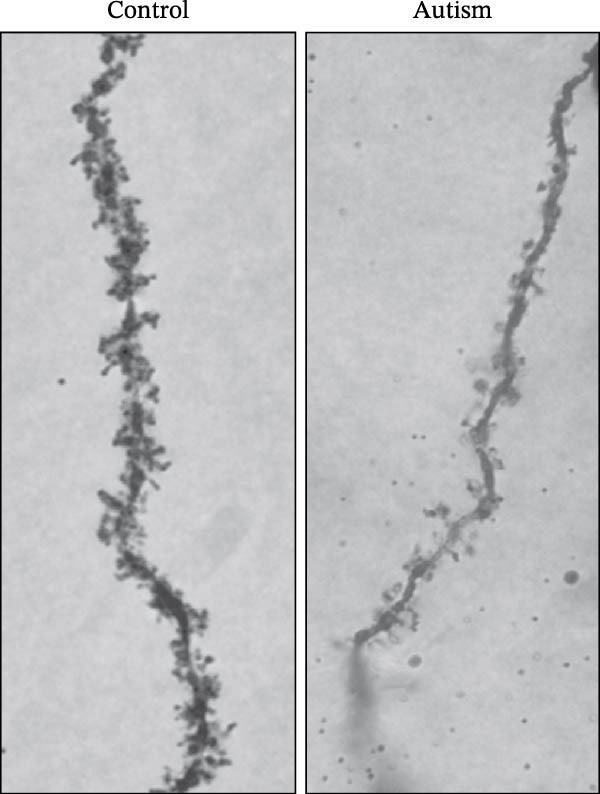
(A)

**Figure:**
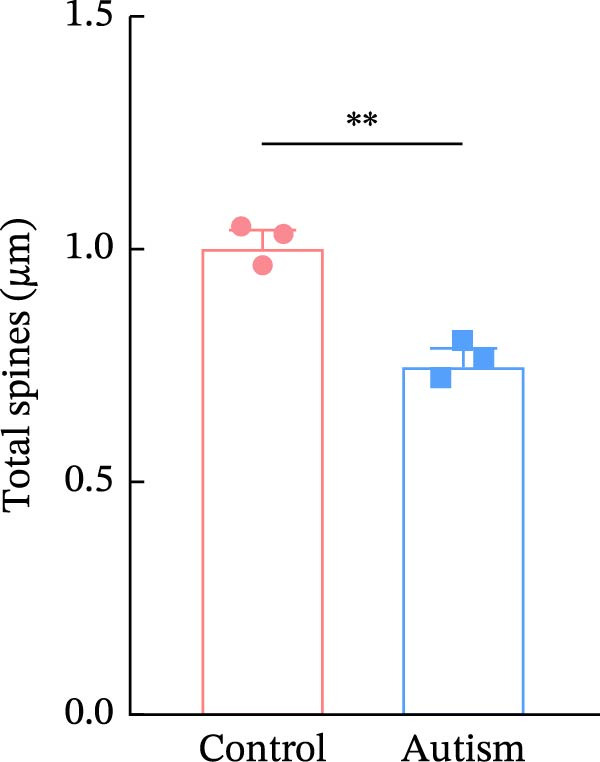
(B)

**Figure:**
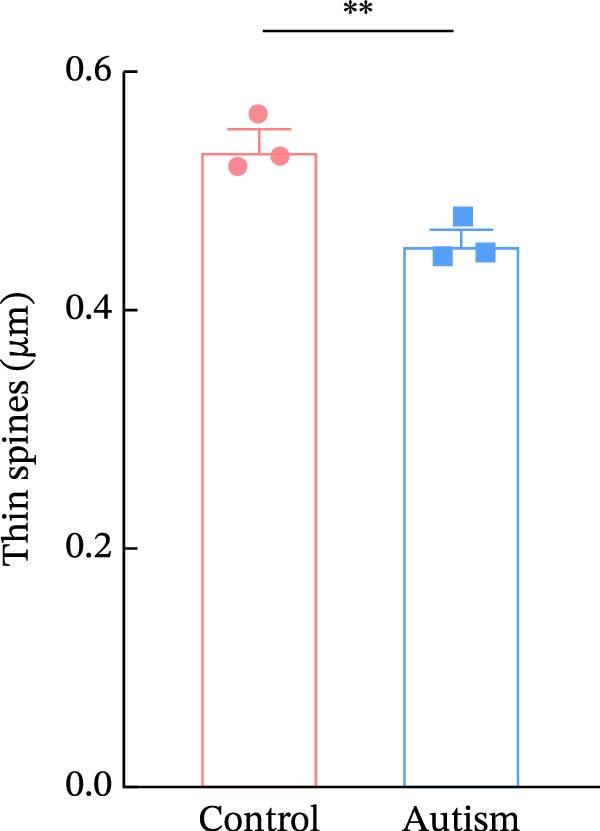
(C)

**Figure:**
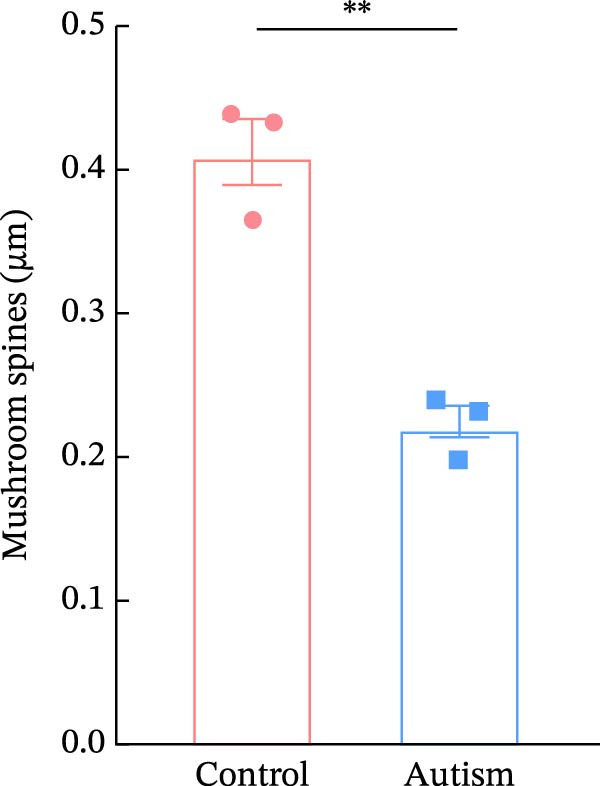
(D)

**Figure:**
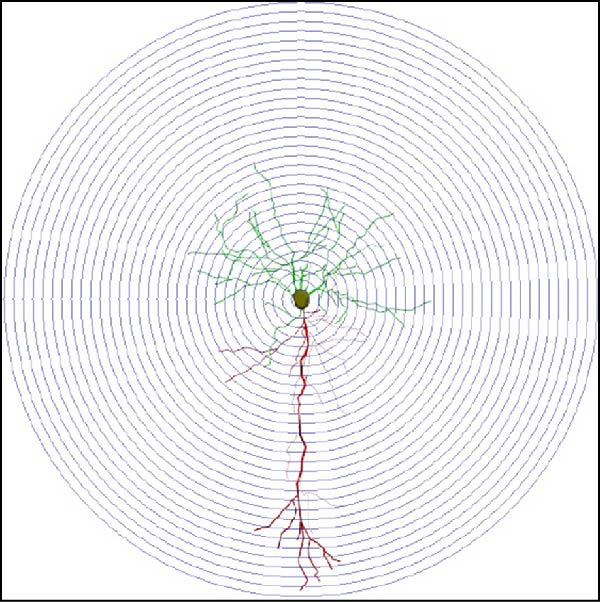
(E)

**Figure:**
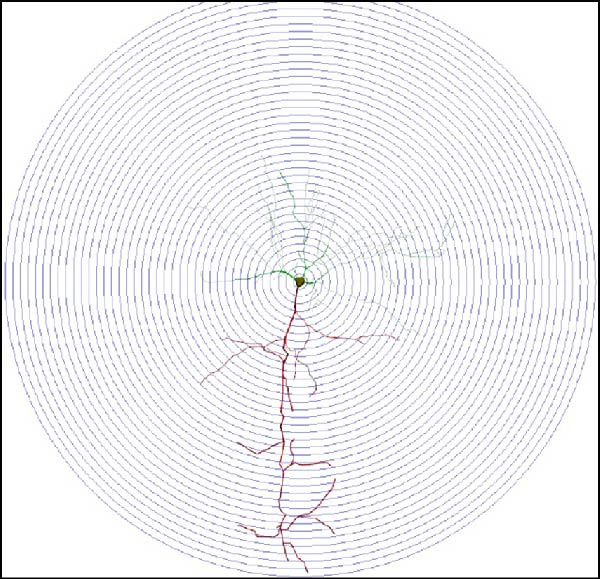
(F)

**Figure:**
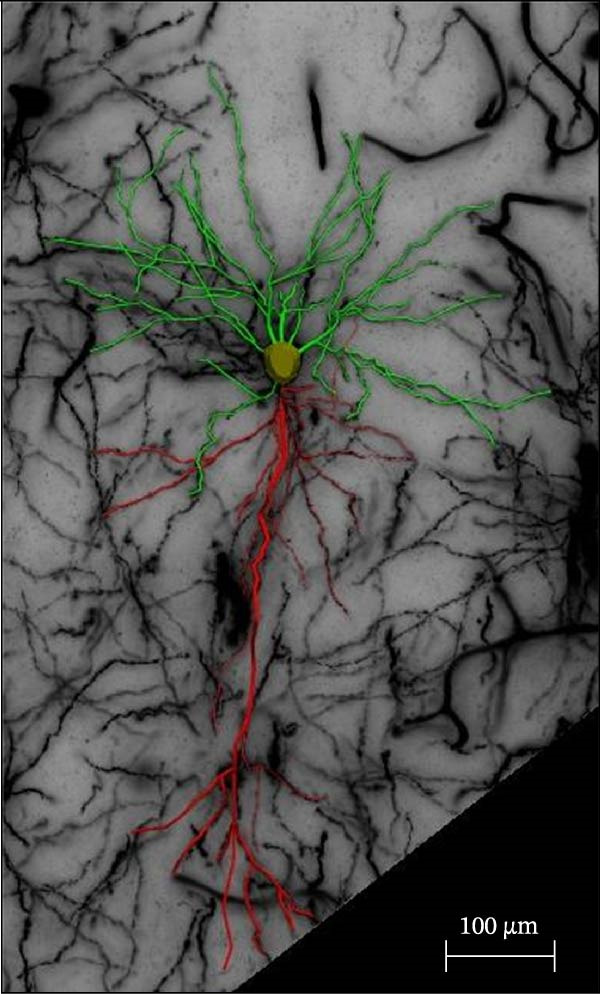
(G)

**Figure:**
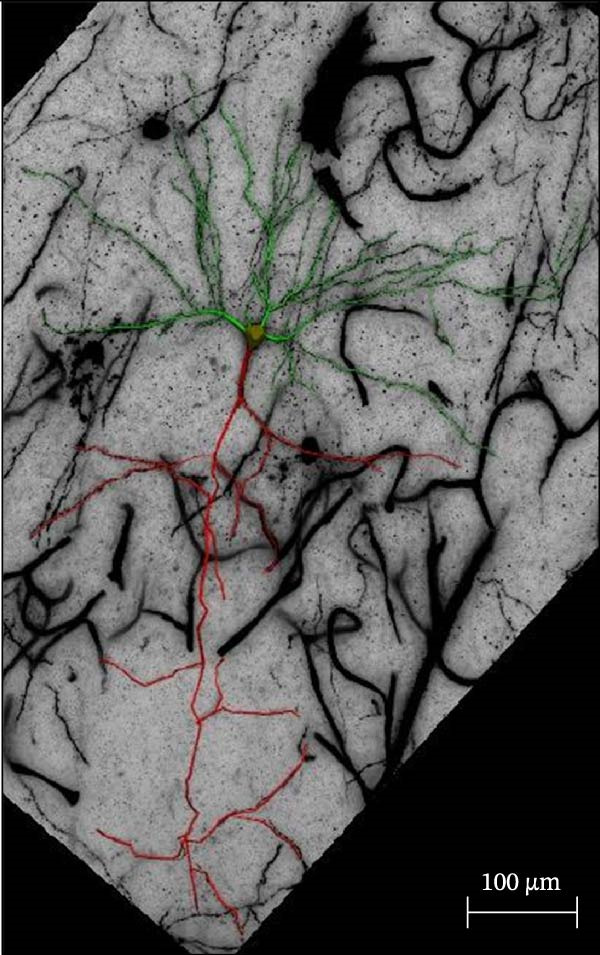
(H)

**Figure:**
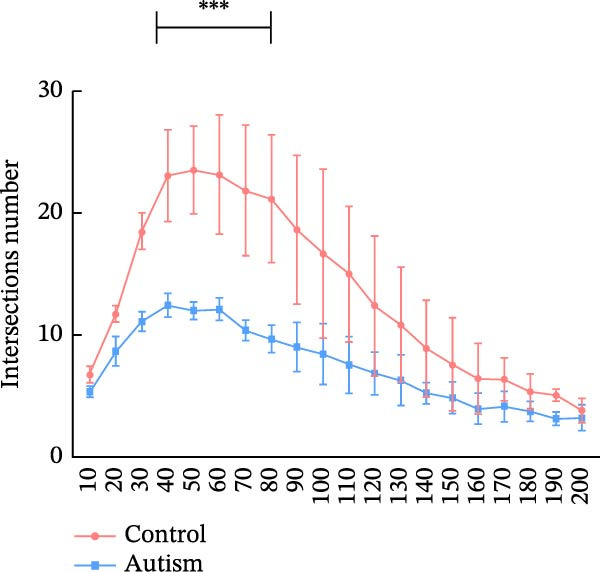
(I)

**Figure:**
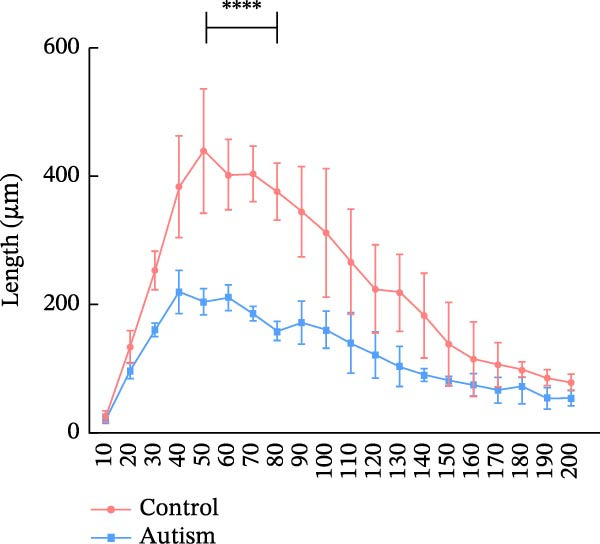
(J)

**Figure:**
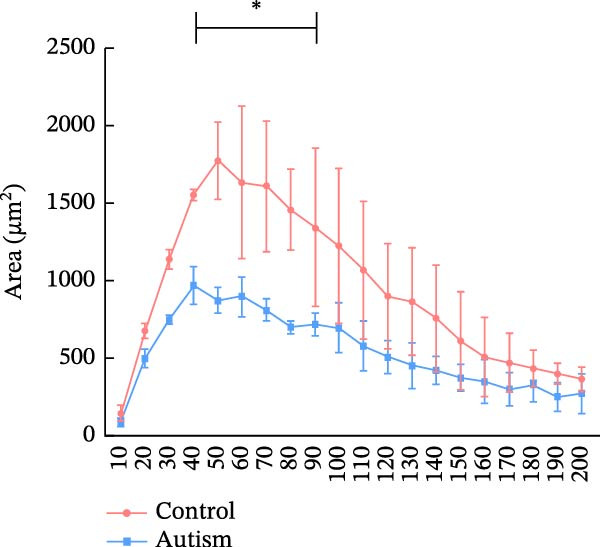
(K)

### 3.2. ASD Rats Exhibited Reduced Expression of 5‐HT1ARs in the PFC

Considering neuronal damage in the PFC caused by VPA exposure, we investigated changes in the expression of 5‐HT1ARs in this region. The qPCR and Western blot analyses of total RNA and protein extracted from the PFC revealed that 5‐HT1AR expression was significantly reduced in ASD rats compared with that in controls (RT‐qPCR: 0.99 ± 0.04 vs. 0.78 ± 0.08, *p* = 0.0147; Western blot: 1.33 ± 0.88 vs. 0.93 ± 0.15, *p* = 0.0302) (Figure 1D–F).

### 3.3. Administration of 5‐HT1AR Agonist Reduced Epilepsy Susceptibility in ASD Rats

To evaluate the susceptibility of ASD rats to PTZ‐induced seizures, both control and ASD model groups were administered intraperitoneal injections of PTZ (60 mg/kg) and subsequently monitored through intracortical EEG for 4 h (Figure 3). Seizure latency, duration of stage IV seizures, and stage IV seizure incidence were assessed based on EEG and video recordings. Fast‐fourier transform (FFT) analyses of EEG data were performed to determine power in the 0–40 Hz frequency range, which indicated higher power in the ASD rats compared with controls.

Figure 3Activation of 5‐HT1A receptors reduced PTZ‐induced seizure activity in ASD rats. (A) Representative EEG traces and corresponding spectrograms showing seizure activity recorded from the prefrontal cortex (PFC) in the control group, ASD model group, ASD + Vehicle group, and ASD + 8‐OH‐DPAT group. (B) Quantification of seizure latency. The ASD model group exhibited significantly shorter seizure latency than the control group, whereas treatment with 8‐OH‐DPAT significantly prolonged seizure latency relative to the ASD + Vehicle group. (C) Total duration of stage IV seizures. The ASD model group exhibited significantly prolonged seizure duration compared with the control group, whereas 8‐OH‐DPAT treatment significantly reduced seizure duration. (D) Incidence of stage IV seizures. The proportion of rats exhibiting stage IV seizures was significantly increased in the ASD model group and was reduced following 8‐OH‐DPAT treatment. (E) Representative Western blot bands showing 5‐HT1A receptor (56 kDa) and GAPDH (37 kDa) in the PFC of the control group, ASD model group, ASD model + Vehicle group, and ASD model + 8‐OH‐DPAT group. (F) Quantitative analysis of 5‐HT1A receptor protein expression normalized to GAPDH. Administration of 8‐OH‐DPAT did not significantly alter total 5‐HT1A receptor protein expression relative to the ASD model + Vehicle group. Data are presented as mean ± SEM (EEG analysis: *n* = 6; Western blot analysis: *n* = 3). Statistical significance was determined using one‐way ANOVA.  ^∗^ *p* < 0.05,  ^∗∗∗^ *p* < 0.001,  ^∗∗∗∗^ *p* < 0.0001.

**Figure:**
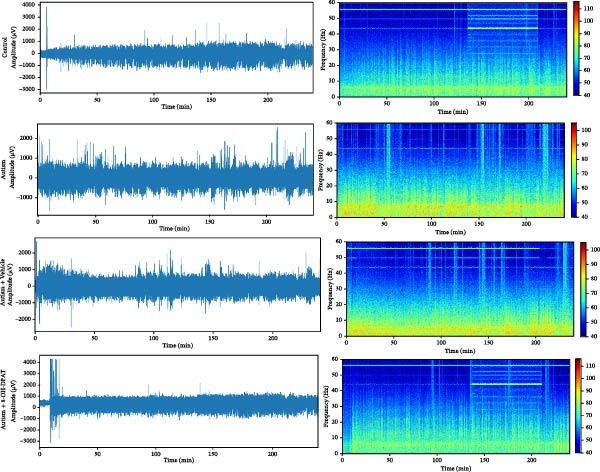
(A)

**Figure:**
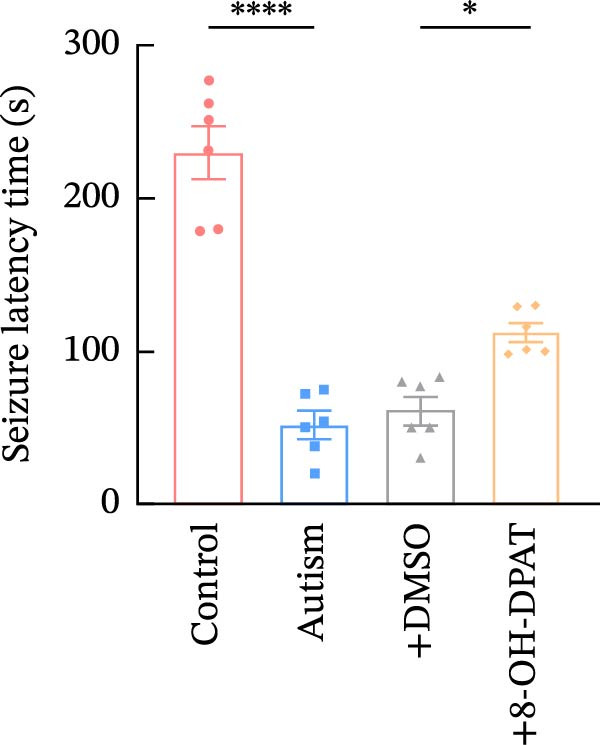
(B)

**Figure:**
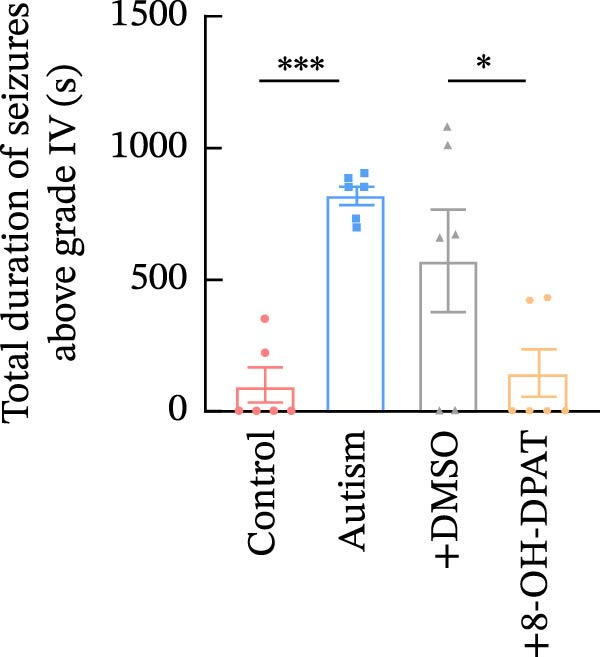
(C)

**Figure:**
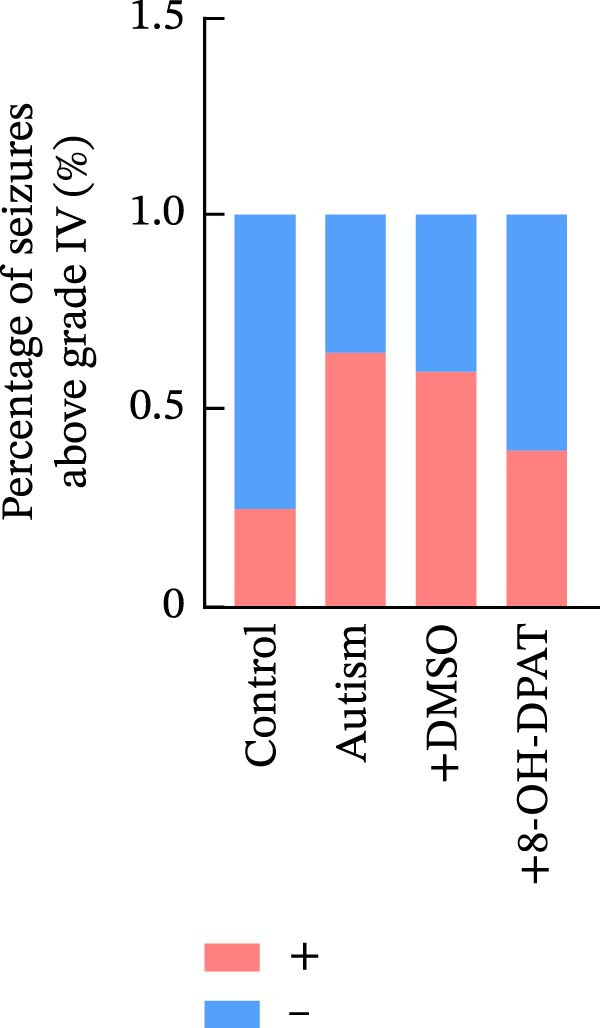
(D)

**Figure:**
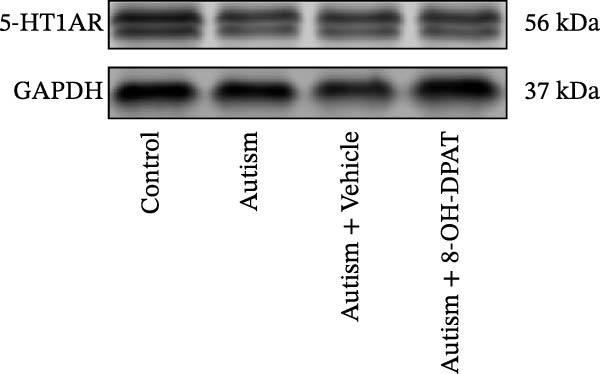
(E)

**Figure:**
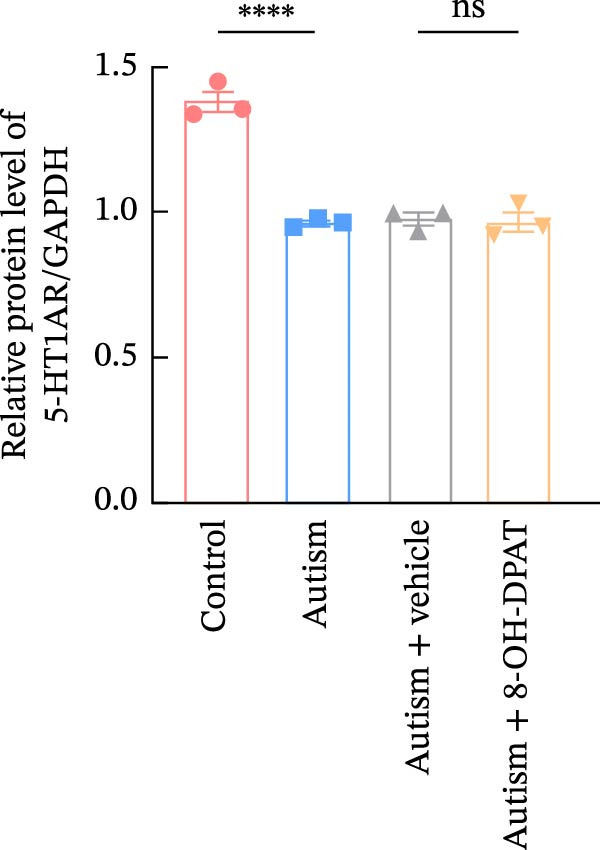
(F)

Compared with the control group, the ASD model group exhibited a significantly shorter latency to seizure onset (51.5 ± 20.76 vs. 229.83 ± 42.14, *p* < 0.0001), prolonged duration of stage IV seizures (217.83 ± 133.28 vs. 819.17 ± 84.62, *p* < 0.0001), and a higher incidence of stage IV seizures (65% vs. 25%, *p* = 0.0110). These results indicated that VPA exposure during the prenatal period increased epilepsy susceptibility in the ASD rats.

To assess the role of 5‐HT1ARs in modulating this susceptibility, the 5‐HT1AR agonist 8‐OH‐DPAT was administered locally to ASD rats through cannulas implanted in their PFC. PTZ (60 mg/kg) was intraperitoneally injected 20 min after 8‐OH‐DPAT administration. Subsequently, EEG monitoring and seizure evaluations were performed. Seizure onset latency was significantly longer in the ASD + 8‐OH‐DPAT group than in the ASD + Vehicle group (112.33 ± 14.76 vs. 61.67 ± 21.45, *p* = 0.0056).

The duration of stage IV seizures in the ASD + 8‐OH‐DPAT group was lower than that in the ASD + Vehicle group; however, the difference was not statistically significant (787.00 ± 201.19 vs. 475 ± 81.67, *p* = 0.2997). Notably, the incidence of stage IV seizures was markedly lower in the ASD + 8‐OH‐DPAT group (65% vs. 25% vs. 60% vs. 40%, *p* = 0.0417).

These results indicated that the local administration of 8‐OH‐DPAT in the PFC reduced the sensitivity to PTZ‐induced seizures in the ASD rats, highlighting the potential role of 5‐HT1AR activation in mitigating epilepsy susceptibility in the ASD model group.

### 3.4. 5‐HT1AR Activation Reduced sAP and mEPSC Frequencies in PFC Pyramidal Neurons

To investigate whether activation of PFC 5‐HT1ARs mitigated PTZ‐induced seizures, whole‐cell patch‐clamp recordings were performed in PFC pyramidal neurons from both control and ASD model groups. Under baseline conditions (without PTZ), the amplitude of sAPs in the ASD model group did not differ significantly from that in the control group (73.58 ± 10.40 vs. 69.38 ± 8.35, *p* = 0.3583); however, the sAP frequency was significantly lower in the ASD model group than in the control group (0.42 ± 0.39 vs. 1.41 ± 0.67, *p* = 0.0014) (Figure 4A–C). Pharmacological interventions were applied to brain slices through the perfusion system. After incorporating PTZ in the perfusion system, the sAP frequency increased significantly in the ASD model group (Figure 4D–F) compared with the control group (14.41 ± 11.69 vs. 5.67 ± 5.41, *p* = 0.032); however, the sAP amplitude insignificantly changed (73.33 ± 3.86 vs. 74.24 ± 5.89, *p* > 0.9999). After administration of 8‐OH‐DPAT, the sAP frequency in the ASD model group decreased significantly (2.36 ± 0.72 vs. 12.36 ± 2.15, *p* = 0.0003) but the sAP amplitude changed insignificantly (73.33 ± 2.56 vs. 70.64 ± 1.64, *p* = 0.77).

Figure 4Effects of 8‐OH‐DPAT on sAP in prefrontal cortex pyramidal neurons. (A) Representative sAP traces under baseline conditions (without PTZ) from the control and ASD model rats. ASD model rats exhibited reduced sAP frequency compared with controls. (B, C) Quantification of sAP frequency (B) and amplitude (C) under baseline conditions. sAP frequency was significantly lower in the ASD model group than in controls, whereas sAP amplitude showed no significant difference between the groups (data are presented as the mean ± SEM, *n* = 3, statistical significance was determined using the independent‐samples *t*‐test.  ^∗^ *p* < 0.05, ns: no significance). (D) Representative sAP traces under PTZ perfusion from the control, ASD model, and ASD model + 8‐OH‐DPAT groups. The ASD model rats displayed increased sAP frequency under PTZ, whereas the ASD + 8‐OH‐DPAT group showed decreased sAP frequency. (E, F) Quantification of sAP frequency (E) and amplitude (F) under PTZ perfusion. The ASD model rats exhibited significantly higher sAP frequency than controls, whereas the ASD + 8‐OH‐DPAT group exhibited significantly reduced sAP frequency. No significant differences in sAP amplitude were observed between the groups (data are presented as the mean ± SEM, *n* = 3, statistical significance was determined using one‐way ANOVA.  ^∗^ *p* < 0.05,  ^∗∗^ *p* < 0.01, ns: no significance).

**Figure:**
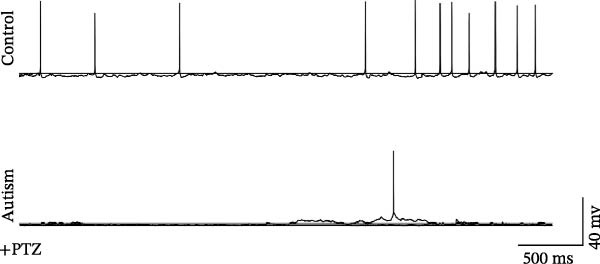
(A)

**Figure:**
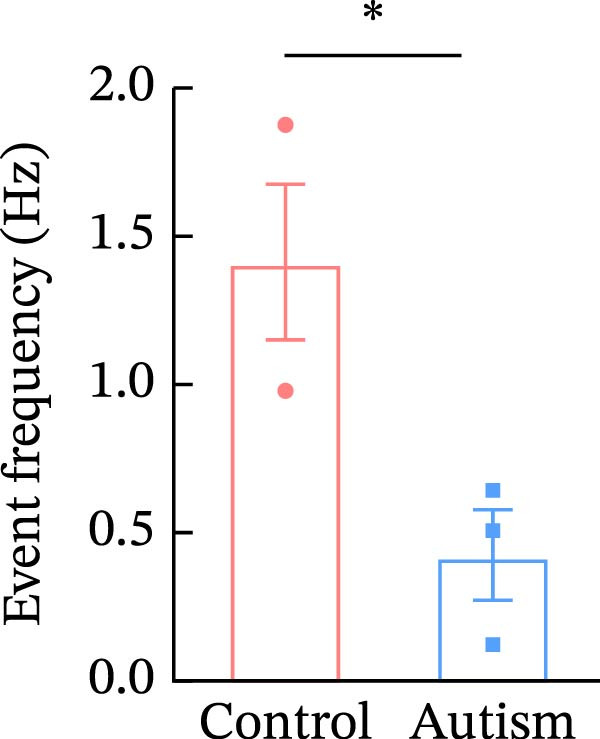
(B)

**Figure:**
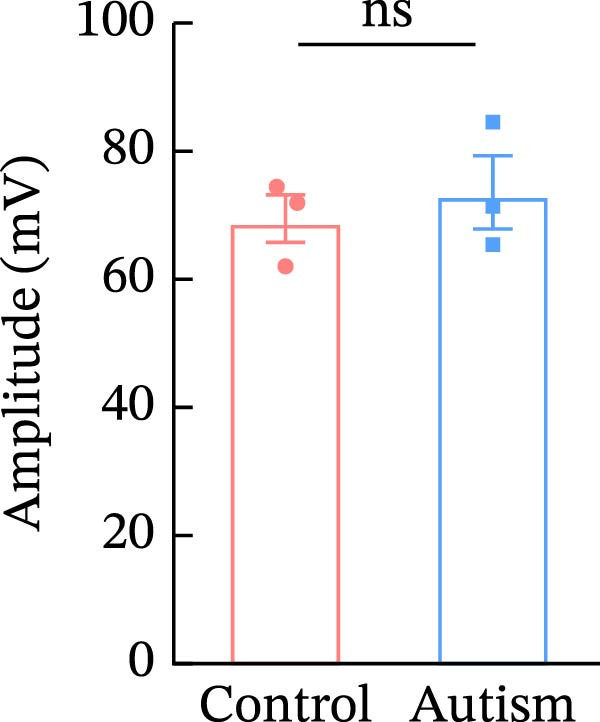
(C)

**Figure:**
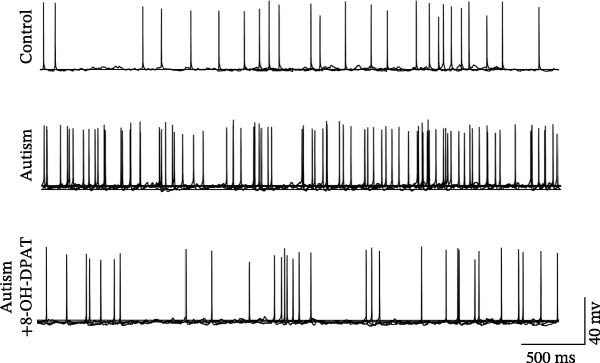
(D)

**Figure:**
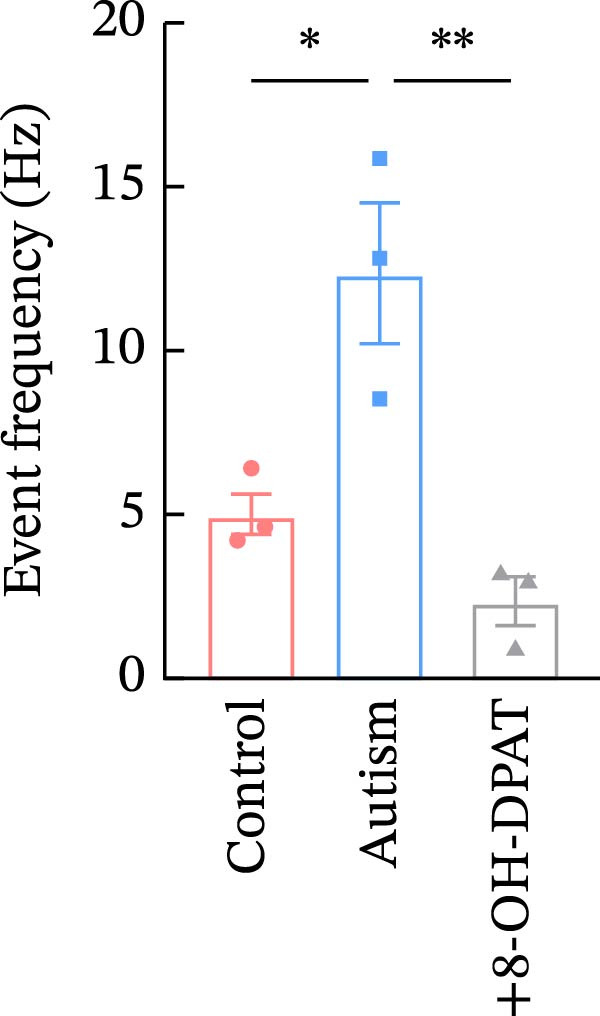
(E)

**Figure:**
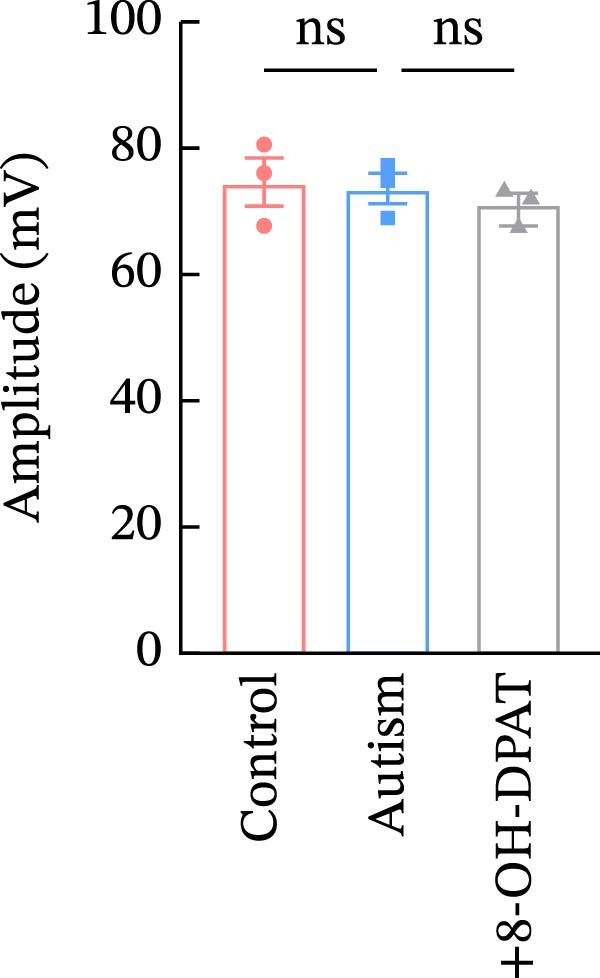
(F)

Subsequent analysis of mEPSCs and mIPSCs revealed that PTZ intervention significantly increased mEPSC frequency in the ASD model group compared with the control group (6.42 ± 1.07 vs. 1.39 ± 0.22, *p* < 0.0001) (Figure 5); however, the mEPSC amplitude, mIPSC frequency, and mIPSC amplitude did not differ significantly between the groups (13.87 ± 2.66 vs. 15.45 ± 4.23, *p* = 0.6211; 0.33 ± 0.16 vs. 0.36 ± 0.18, *p* > 0.9999; 12.34 ± 5.03 vs. 11.22 ± 0.60, *p* = 0.7687, respectively).

Figure 5Effects of 8‐OH‐DPAT on miniature inhibitory and excitatory postsynaptic currents in prefrontal cortex pyramidal neurons. (A) Representative miniature inhibitory postsynaptic current (mIPSC) traces from the control, ASD model, and ASD + 8‐OH‐DPAT groups. (B–E) Quantification of mIPSC frequency (B) and cumulative probability plot of mIPSC interevent intervals (C); quantification of mIPSC amplitude (D), and cumulative probability plot of mIPSC amplitude (E). No significant differences were observed in mIPSC frequency or amplitude among the groups. (F) Representative miniature excitatory postsynaptic current (mEPSC) traces under PTZ perfusion from the control, ASD model, and ASD + 8‐OH‐DPAT groups. (G–J) Quantification of mEPSC frequency (G) and cumulative probability plot of mEPSC interevent intervals (H); quantification of mEPSC amplitude (I) and cumulative probability plot of mEPSC amplitude (J). The ASD model group exhibited significantly higher mEPSC frequency than controls; however, the ASD + 8‐OH‐DPAT group showed significantly reduced mEPSC frequency, and mEPSC amplitude did not differ significantly among the groups (data are presented as the mean ± SEM, *n* = 3, statistical significance was determined using one‐way ANOVA.  ^∗∗∗∗^ *p* < 0.0001, ns: no significance).

**Figure:**
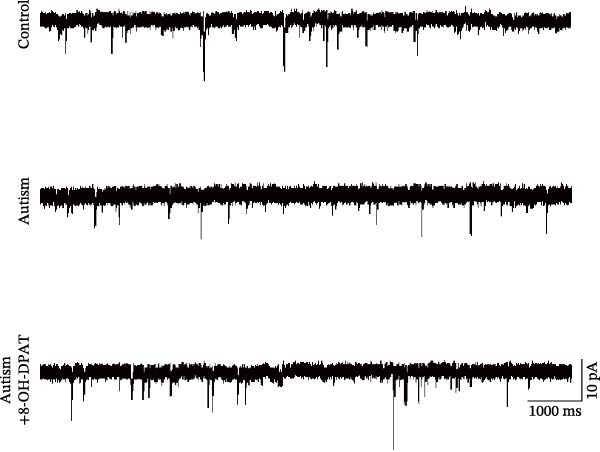
(A)

**Figure:**
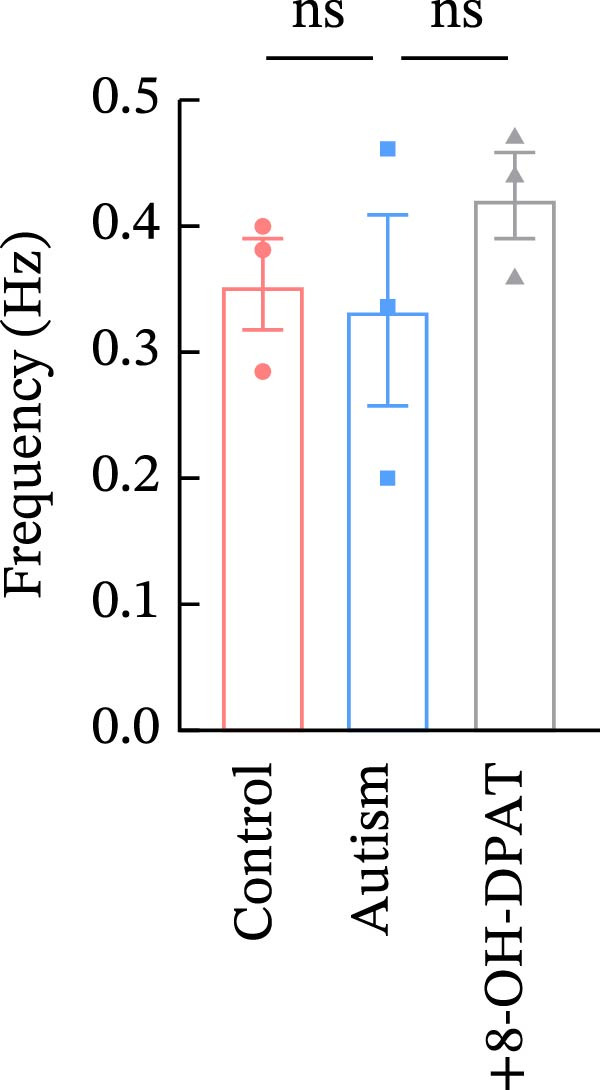
(B)

**Figure:**
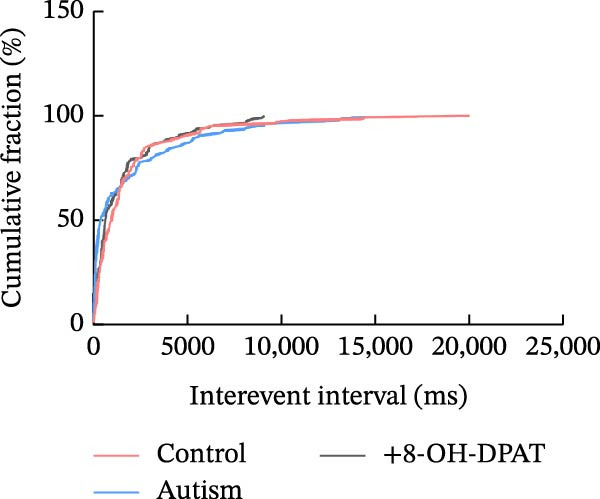
(C)

**Figure:**
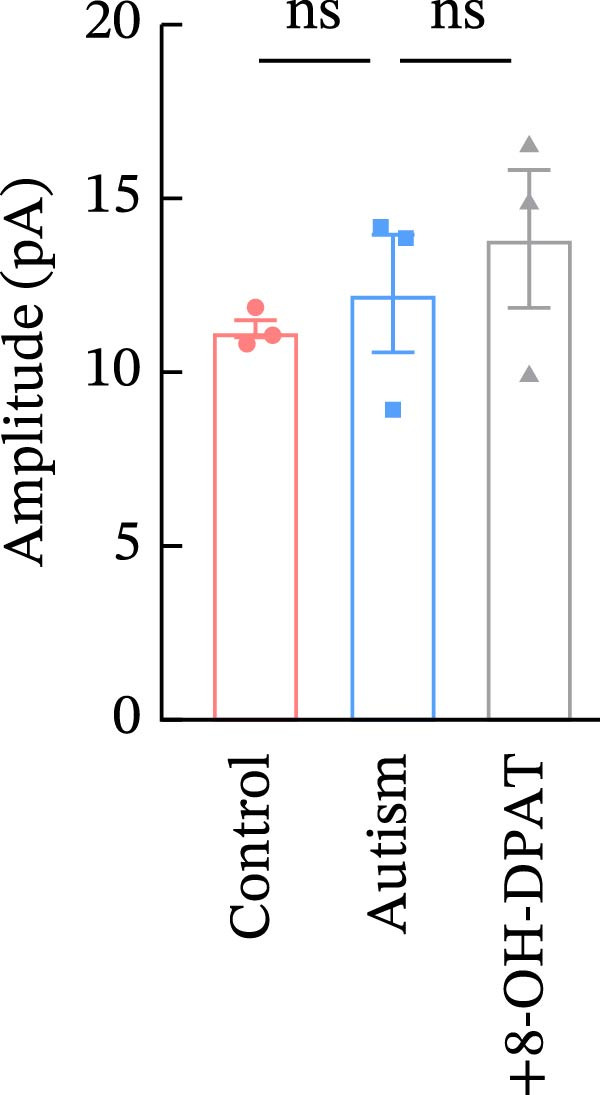
(D)

**Figure:**
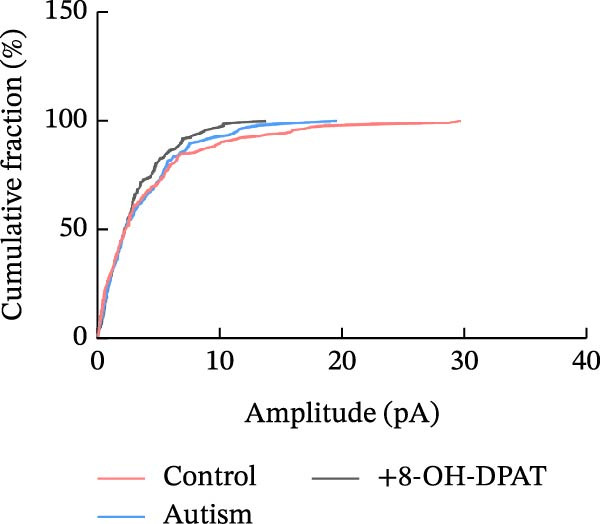
(E)

**Figure:**
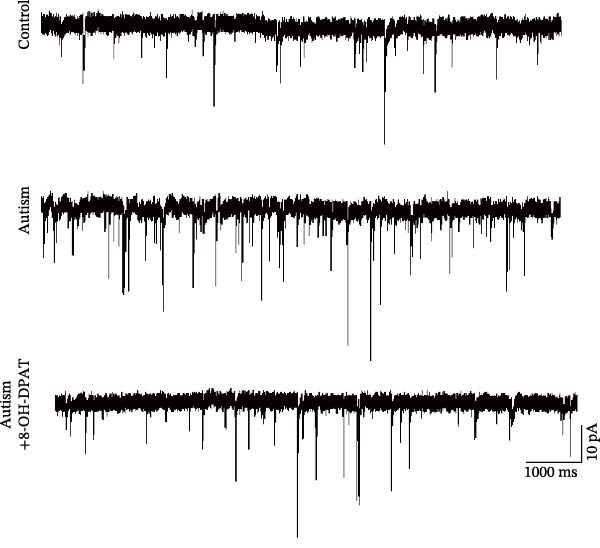
(F)

**Figure:**
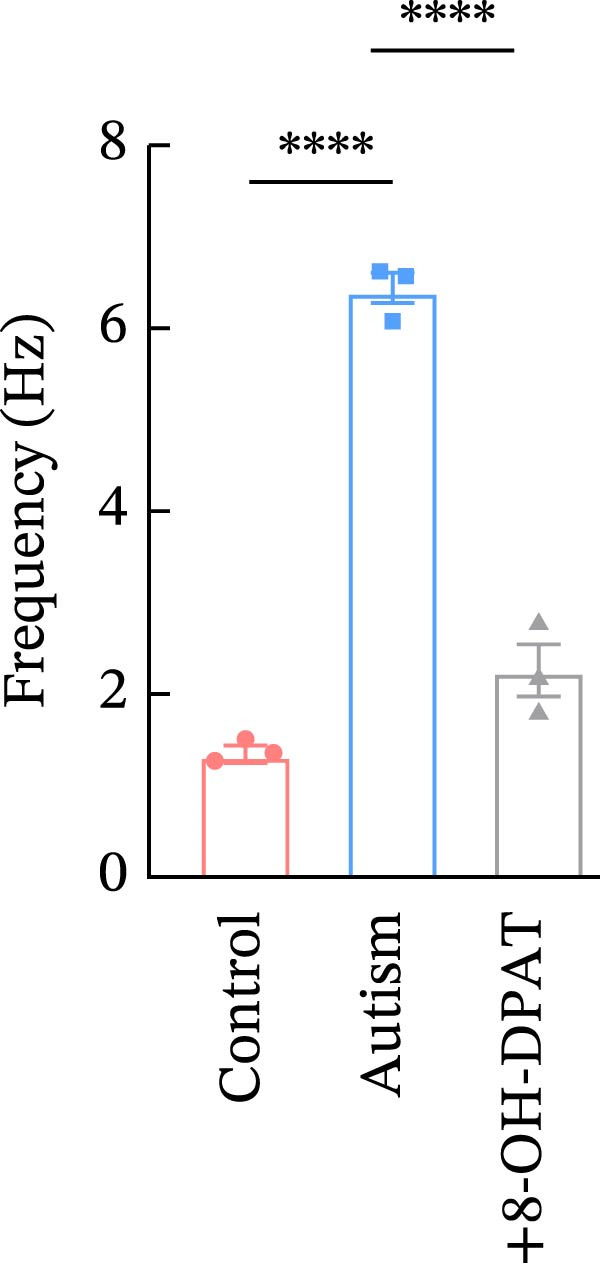
(G)

**Figure:**
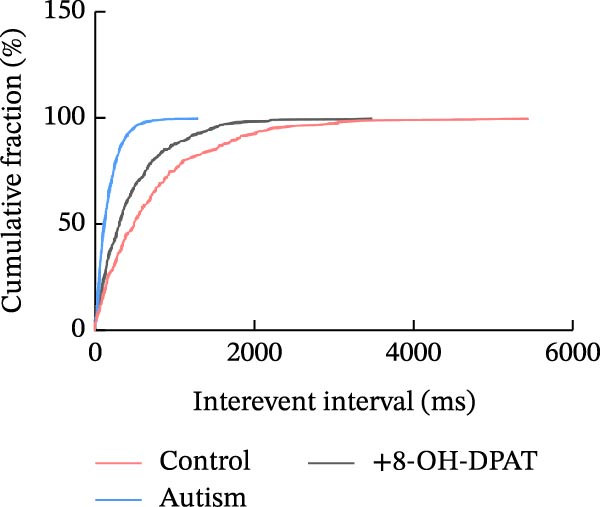
(H)

**Figure:**
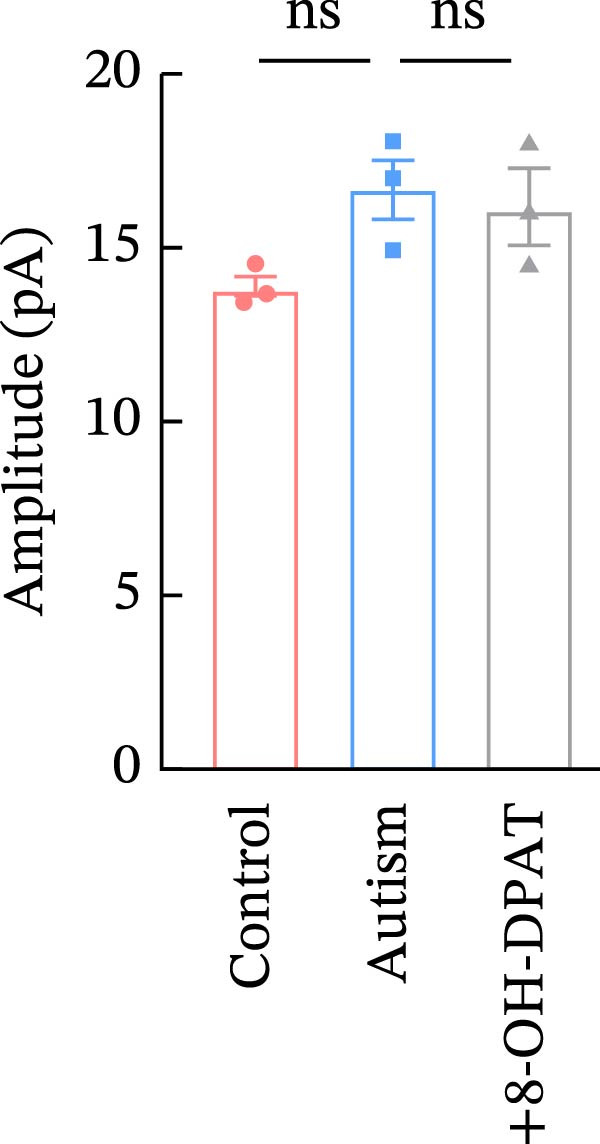
(I)

**Figure:**
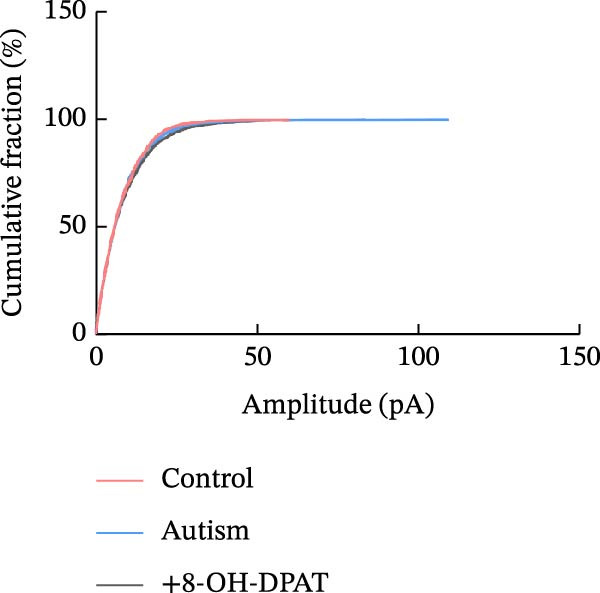
(J)

In the ASD + 8‐OH‐DPAT group (with PTZ), both sAP and mEPSC frequencies decreased significantly (2.56 ± 1.86 vs. 14.41 ± 11.69, *p* = 0.0003; 2.26 ± 1.39 vs. 6.42 ± 1.07, *p* < 0.0001); however, sAP amplitude, mEPSC amplitude, and mIPSC frequency and amplitude were not significantly different compared with those of the ASD + Vehicle group (70.64 ± 3.03 vs. 73.33 ± 3.86, *p* = 0.2716; 17.36 ± 3.61 vs. 13.87 ± 2.66, *p* = 0.1153; 0.43 ± 0.11 vs. 0.33 ± 0.16, *p* > 0.9999; 13.85 ± 3.11 vs. 12.34 ± 5.03, *p* = 0.6255). These results indicated that 8‐OH‐DPAT‐induced activation of 5‐HT1AR reduced the excitability of PFC pyramidal neurons, as evidenced by decreased sAP and mEPSC frequencies observed in the ASD model, potentially contributing to antiepileptic effects.

### 3.5. Activation of 5‐HT1AR‐Coupled Kir3 Channels Modulated PFC Pyramidal Neuron Excitability

To assess ionic conductance in the PFC pyramidal neurons, we recorded the postsynaptic excitability after blocking AMPA receptors (AMPARs), NMDA receptors (NMDARs), GABAA receptors (GABAARs), metabotropic glutamate receptors (mGluRs), and voltage‐gated sodium channels by using 10 μM DNQX, 50 μM AP‐5, 1 μM gabazine, 10 μM LY‐367385, 1 μM CGP‐55845, and 1 μM TTX, respectively. Whole‐cell voltage‐clamp recordings were performed on the cells during the hyperpolarizing and depolarizing steps; finally, the I–V curves were generated (see Methods for details).

In pyramidal neurons from the rats treated with 8‐OH‐DPAT, inwardly rectifying current was observed, with the reversal potential close to the value observed for potassium channels (−85 mV). This inward rectification was absent in neurons from the untreated rats. The inwardly rectifying current was quantified by calculating the ratio of current responses at −60 mV to those at −110 mV, termed the rectification index (RI). Pyramidal neurons in the untreated ASD model group displayed a significantly lower RI than those in the group treated with 8‐OH‐DPAT (−0.11 ± 0.06 vs. −0.51 ± 0.01, *p* = 0.0021).

These findings suggested that 5‐HT1AR‐induced inwardly rectifying potassium currents mediate the hyperpolarization of pyramidal neurons and the subsequent reduction in firing. To test this hypothesis, we applied the toxin tertiapin‐Q (TQ) to block Kir3 channels, which attenuated the changes induced by 8‐OH‐DPAT and led to a significant reduction in the RI (−0.51 ± 0.01 vs. −0.12 ± 0.06, *p* = 0.0173) (Figure 6).

Figure 6Activation of 5‐HT1A receptors induced inwardly rectifying potassium (Kir3) currents in prefrontal cortex pyramidal neurons. (A, B) Current responses to voltage steps in the ASD group (A) and ASD + 8‐OH‐DPAT group (B); inward rectifying current was observed only in the ASD + 8‐OH‐DPAT group. (C) Schematic of the voltage step protocol used to generate I–V curves, with steps from −110 to −30 mV achieved in 10mV increments. (D) I–V curves showing membrane current responses in the ASD model rats under different conditions: without treatment (ASD group), after 8‐OH‐DPAT treatment (+8‐OH‐DPAT), and after 8‐OH‐DPAT treatment in the presence of the Kir3 channel blocker tertiapin‐Q (+8‐OH‐DPAT + TP‐Q); treatment with 8‐OH‐DPAT significantly induced inward rectification, which was attenuated in the presence of TP‐Q. (E) Determination of the rectification index (RI), calculated as the ratio of current responses at −60 mV to those at −110mV. The RI was significantly lower in the 8‐OH‐DPAT‐treated group than in the untreated ASD model rats but returned to baseline levels in the presence of TP‐Q (data are presented as the mean ± SEM, *n* = 3, statistical analysis was performed using one‐way ANOVA.  ^∗∗^ *p* < 0.01).

**Figure:**
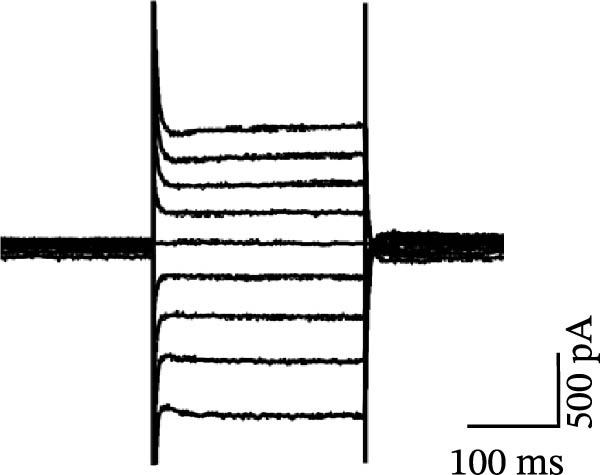
(A)

**Figure:**
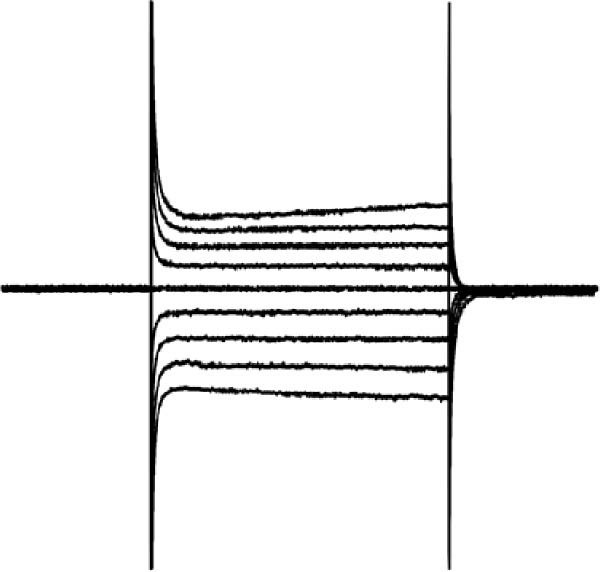
(B)

**Figure:**
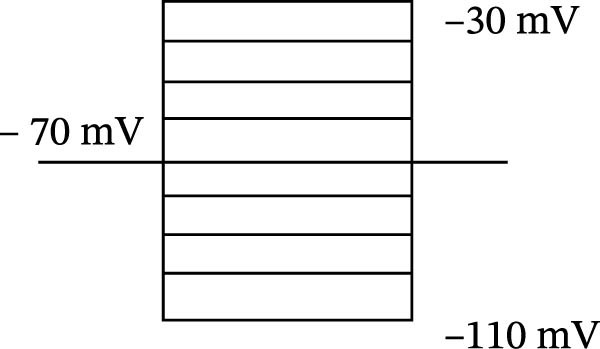
(C)

**Figure:**
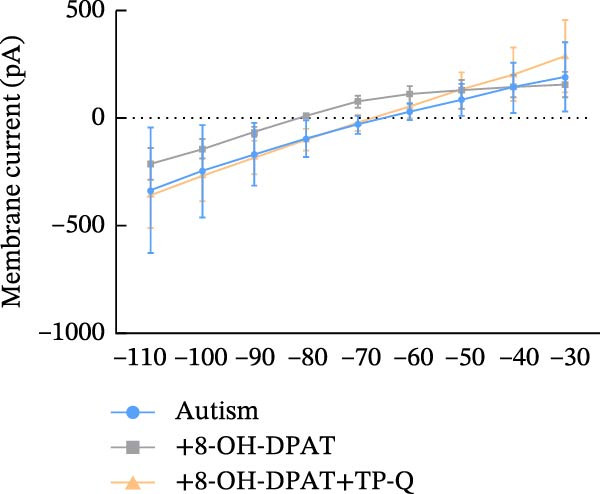
(D)

**Figure:**
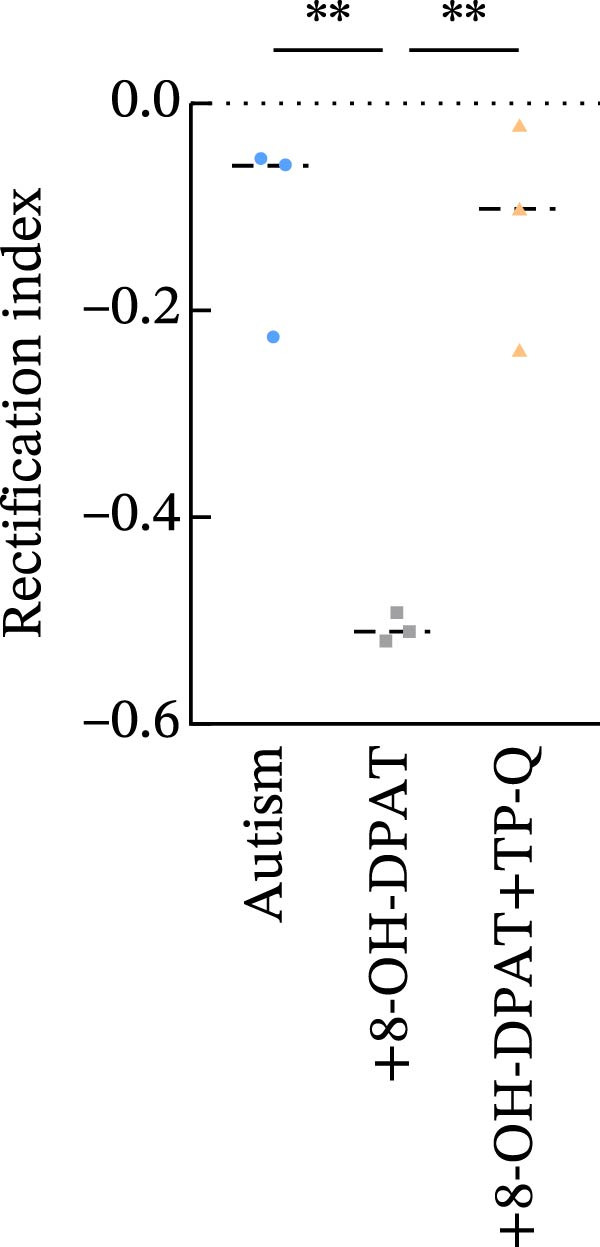
(E)

### 3.6. TP‐Q Attenuated the 8‐OH‐DPAT‐Induced Decrease in sAP and mEPSC Frequencies

The effects of TP‐Q, the Kir3 channel blocker, were determined on sAP, mIPSC, and mEPSC in all experimental groups to confirm the role of Kir3 channels in the excitability of PFC pyramidal neurons. The ASD rats treated with 8‐OH‐DPAT demonstrated reduced sAP frequency, which rebounded following the administration of TP‐Q (2.13 ± 0.3 vs. 7.38 ± 0.94, *p* = 0.0482) (Figure 7A). Neither drug had a significant effect on the amplitude of sAP (71.9 ± 2.42 vs. 73.44 ± 3.6, *p* = 0.7767) (Figure 7B) and the frequency of mIPSC (0.45 ± 0.1 vs. 0.43 ± 0.04, *p* > 0.9999); however, both 8‐OH‐DPAT and TP‐Q had a similar effect on mIPSC (Figure 7C). Although both 8‐OH‐DPAT and TP‐Q led to an increase in mIPSC amplitude in the ASD rats, the increase was not statistically significant compared with untreated groups (11.68 ± 0.79 vs. 11.70 ± 2.11, *p* > 0.9999) (Figure 7D); 8‐OH‐DPAT decreased the frequency of mEPSC, whereas TP‐Q increased it (3.28 ± 0.34 vs. 6.25 ± 0.2, *p* = 0.0006) (Figure 7E). The amplitude of mEPSC did not differ significantly (18.69 ± 0.96 vs. 19.16 ± 1.54, *p* = 0.9019) (Figure 7F). These results demonstrated that specific blocking of the Kir3 channels altered the excitability of pyramidal neurons in the PFC of ASD rats.

Figure 7Changes in the spontaneous action potential, miniature inhibitory postsynaptic current, and miniature excitatory postsynaptic current in each group after specific inhibition of Kir3 channels. (A, B) Frequency and amplitude of the spontaneous action potential (sAP) from PFC pyramidal neurons in the rats before and after the administration of the Kir3 channel blocker. (C, D, G, H) Frequency and amplitude of mIPSC from PFC pyramidal neurons in the rats before and after the administration of the Kir3 channel blocker. (E, F, I, J) Frequency and amplitude of mEPSC from PFC pyramidal neurons in the rats before and after the administration of the Kir3 channel blocker (data are presented as the mean ± SEM, *n* = 3 biologically independent rats, statistical analysis was performed using one‐way ANOVA.  ^∗^ *p* < 0.05,  ^∗∗^ *p* < 0.01,  ^∗∗∗^ *p* < 0.001, ns: no significance).

**Figure:**
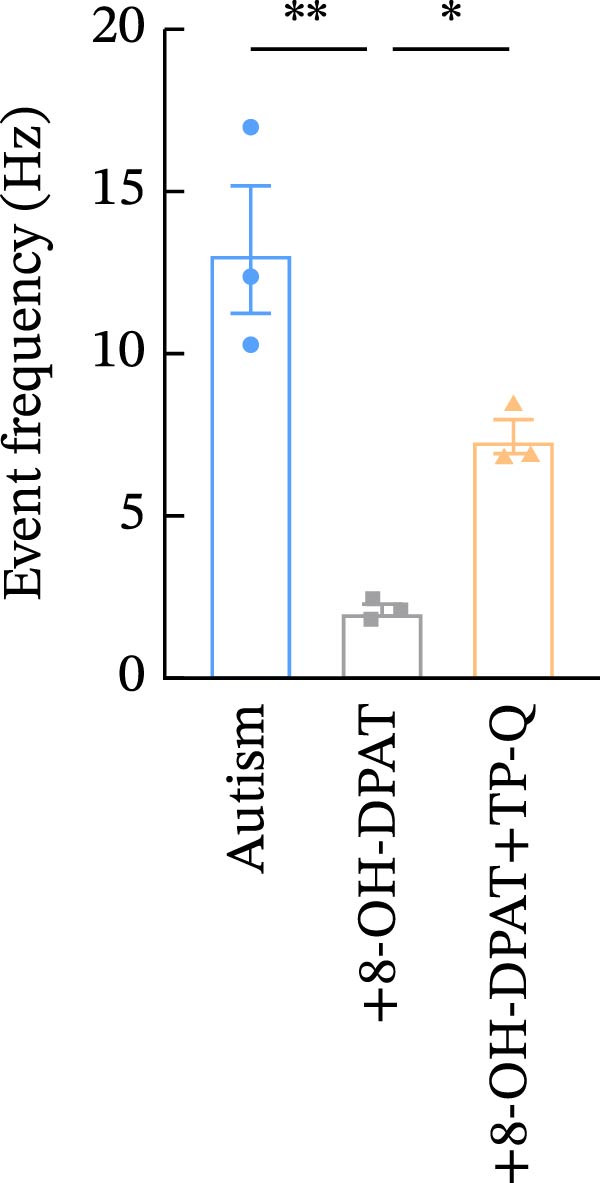
(A)

**Figure:**
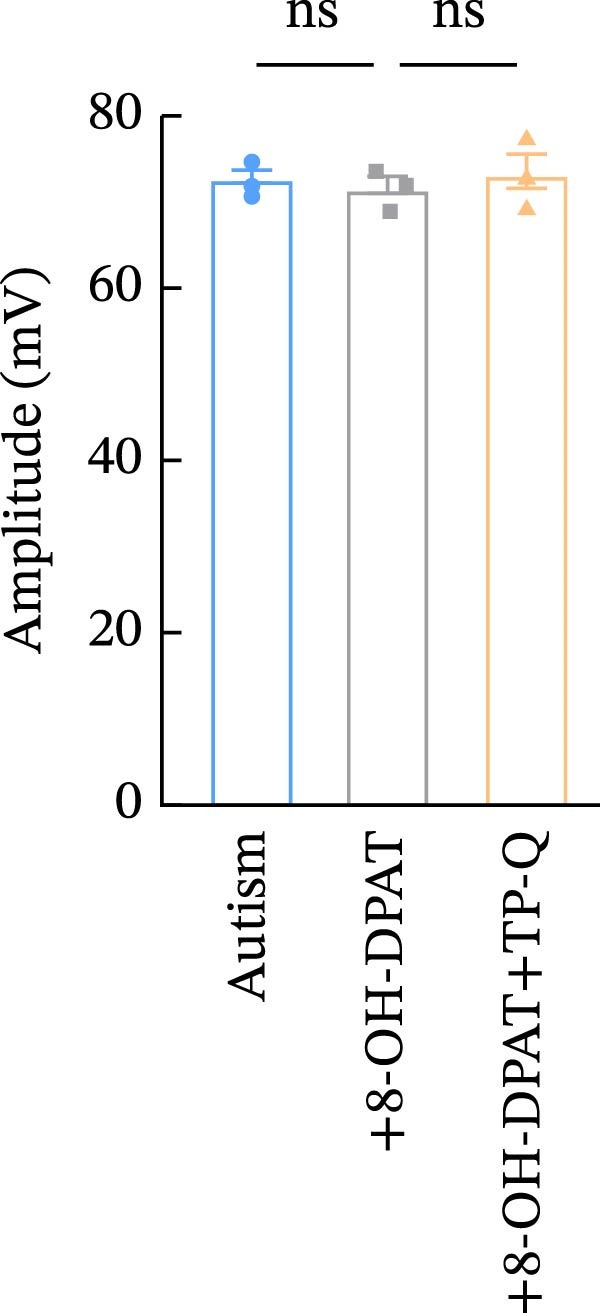
(B)

**Figure:**
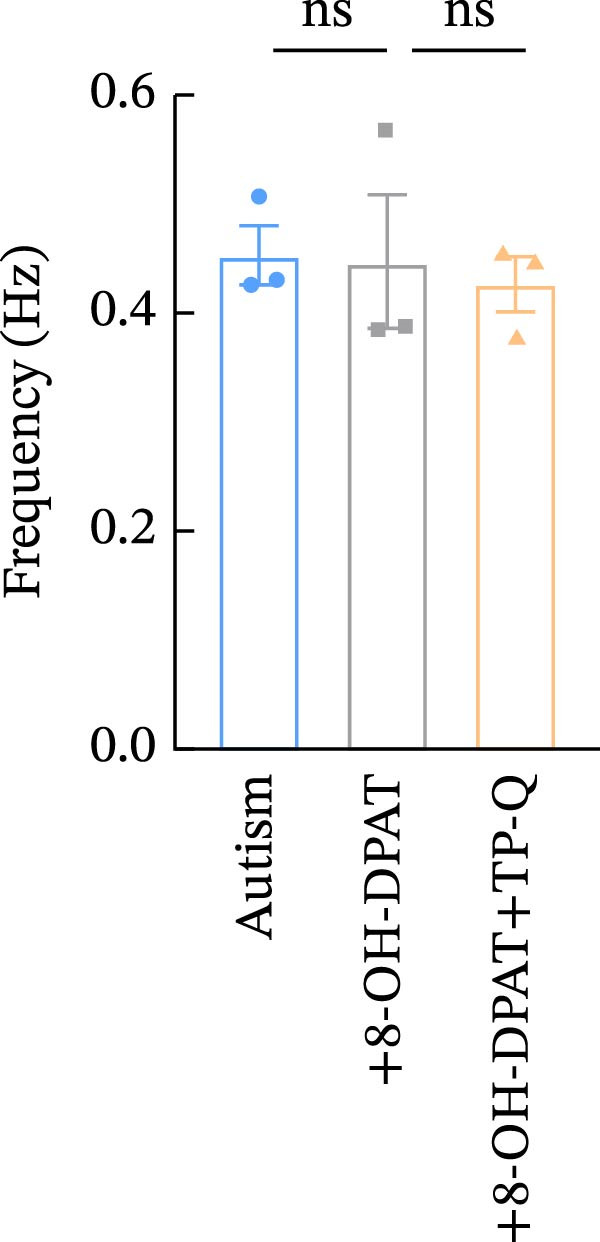
(C)

**Figure:**
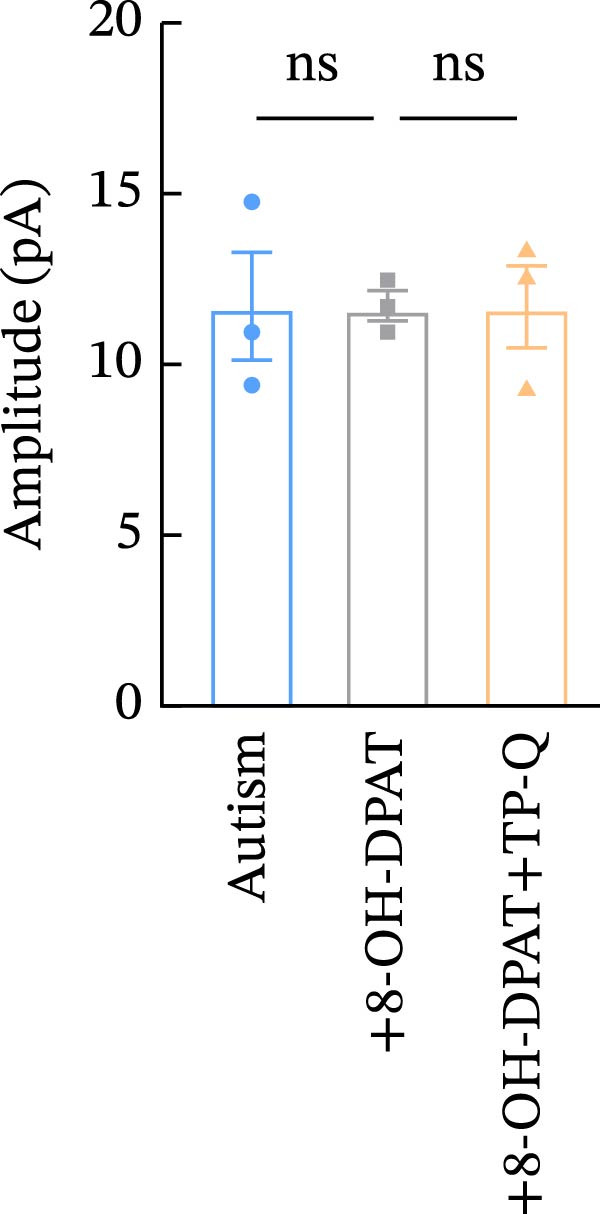
(D)

**Figure:**
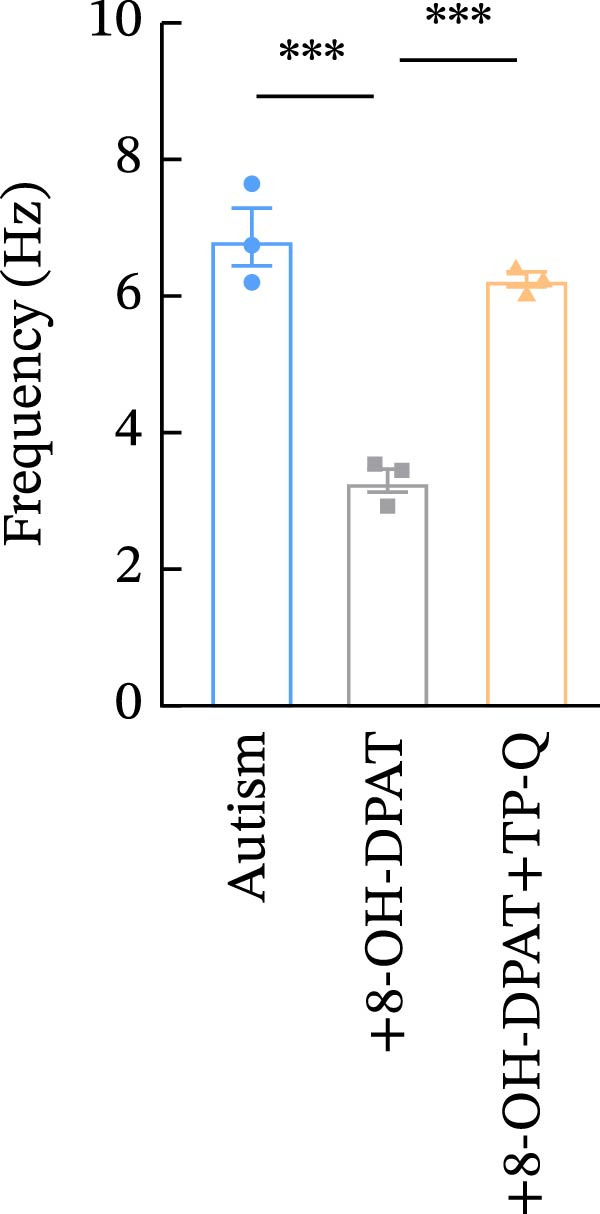
(E)

**Figure:**
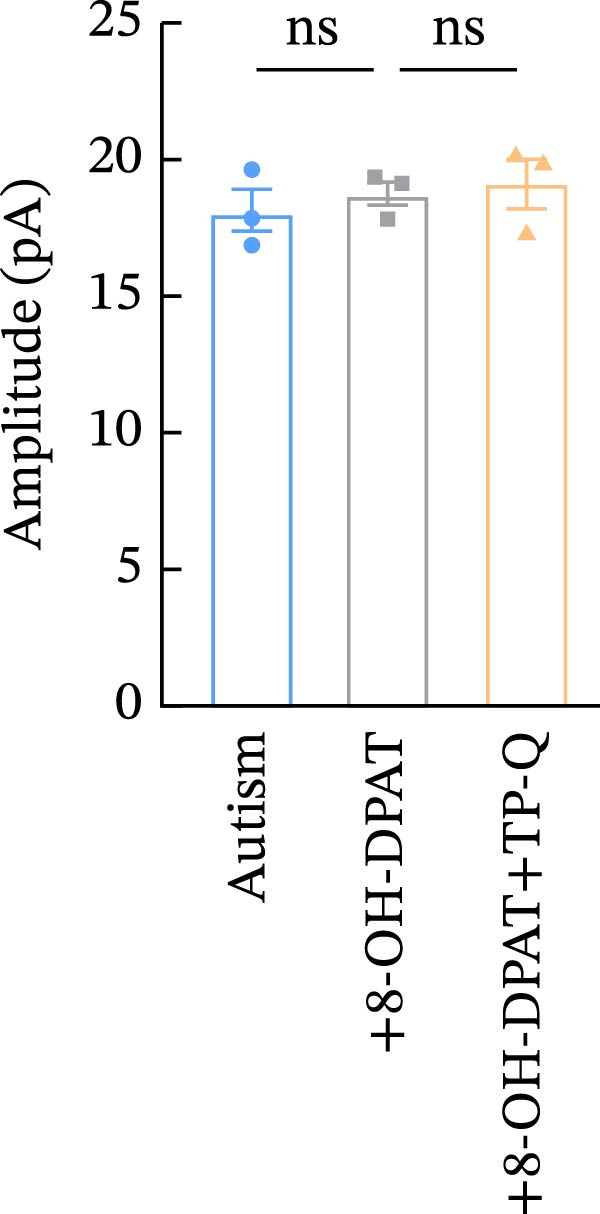
(F)

**Figure:**
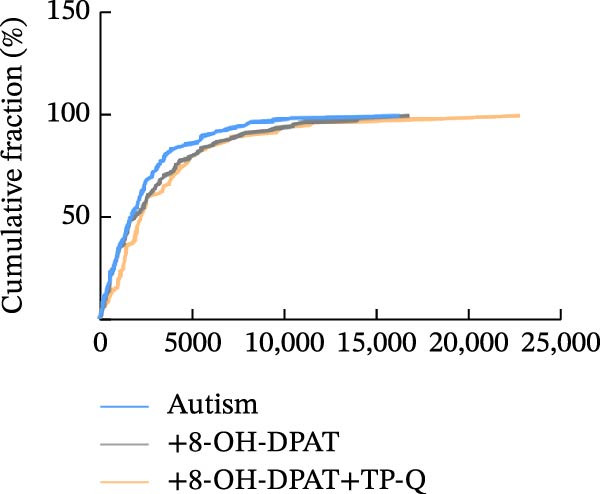
(G)

**Figure:**
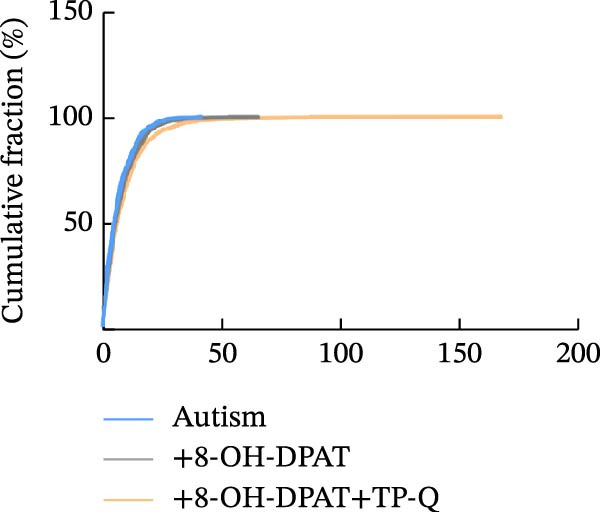
(H)

**Figure:**
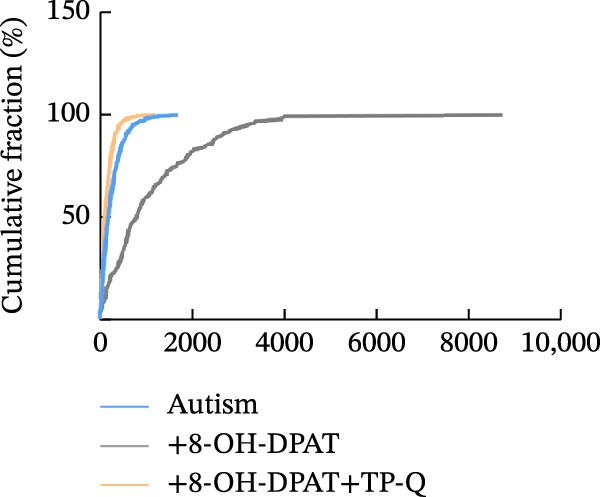
(I)

**Figure:**
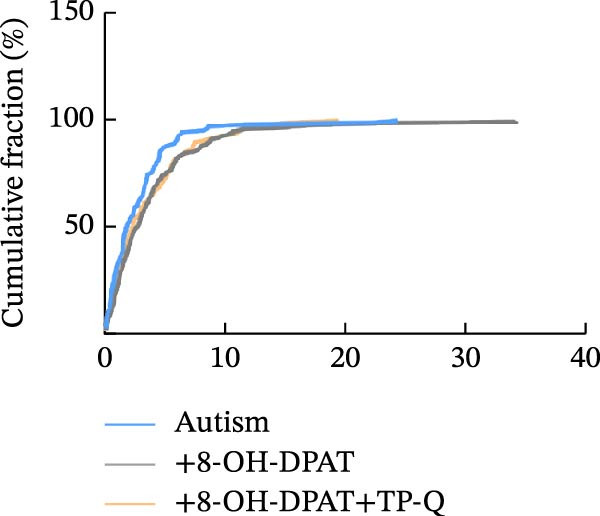
(J)

Overall, the results underscored the role of Kir3 channels in mediating the effects of 5‐HT1AR activity on PFC pyramidal neuron excitability, providing mechanistic insights into the inhibitory effects of 5‐HT1AR agonists.

## 4. Discussion

Epilepsy and ASD share common pathological features characterized by dendritic abnormalities, particularly reduced dendritic branching complexity and altered dendritic spine morphology. In the present study, the VPA‐induced ASD rat model demonstrated abnormal neuronal development in the PFC and an increased susceptibility to epilepsy. Our results indicate that the activation of the 5‐HT1AR in the PFC reduced epilepsy susceptibility through activation of Kir3 channels. These findings align with and expand the existing literature, offering insights into the shared mechanism underlying ASD and epilepsy.

### 4.1. Developmental Deficits in the PFC of ASD Rats

In a study on the medial PFC of mice, a subpopulation of PFC neurons exhibited increased activity upon perceiving unfamiliar animals, but not upon interactions with inanimate objects or empty chambers [31]. In addition, bilateral damage to PFC neurons is known to reduce social functions [32]. Therefore, the PFC has garnered increasing attention, particularly in ASD research. According to developmental trajectories of the PFC in humans, synaptic gene expression in the PFC peaks in adolescence to early puberty, which is a critical time window for phenotypic expression, diagnosis, and treatment of children with ASD, epilepsy, and intellectual disabilities [33]. The PFC is critical for higher‐order cognitive, emotional, and social functions [34], and its dysfunction is strongly associated with ASD [35]. Our study found that PFC neurons in ASD rats exhibited excessive cellular crumpling, nuclear fragmentation, and lysis, a significantly reduced number of positive cells with intact Nissl body structures, reduced neuronal soma area, reduced dendritic complexity, and reduced dendritic spine density. These findings are consistent with those of previous studies reporting that the PFC of individuals with ASD exhibits structural abnormalities, including dendritic overgrowth and subsequent pruning defects [33]. Our results also indicated reduced neuronal soma area in the PFC of ASD rats, although the decrease was not statistically significant, consistent with the results of a previous study [36] and Nissl staining. Reduced neuronal soma area in the PFC potentially contributed to the impairments in working memory and social information integration, consequently overamplifying sensory inputs, which might have increased the susceptibility of epilepsy in ASD rats. Additionally, the reductions in dendritic complexity and spine density potentially impaired synaptic transmission, contributing to behavioral deficits as well as increased seizure susceptibility associated with ASD [37, 38]. In the present study, stubby spine density did not show a significant difference among groups. This result is plausible because stubby spines typically account for a relatively small fraction of dendritic spines in adolescent and adult rodents, and their functional role is less clearly defined than that of thin and mushroom spines. In contrast, the observed reductions in thin and mushroom spine density are likely to have greater implications for synaptic plasticity in PFC pyramidal neurons. Thin spines are generally considered highly dynamic structures that support synaptic remodeling and experience‐dependent plasticity, whereas mushroom spines usually represent more stable and functionally potent excitatory synapses [39]. Therefore, the simultaneous reduction in these two spine subtypes in the ASD model group suggests both impaired synaptic plasticity and a loss of mature excitatory synaptic connectivity within the PFC. Importantly, this finding extends previous observations by suggesting that the model affects not only total spine number but also the relative distribution of spine populations associated with distinct structural and functional states. Consistent with this interpretation, selective loss of thin spines has been associated with compromised cortical plasticity and cognitive dysfunction, particularly in the PFC, whereas reductions in mushroom spines may reflect destabilization of established synaptic networks [40, 41]. Collectively, these alterations in dendritic spine morphology may provide a structural basis for the PFC‐dependent behavioral deficits observed in this model.

Similarly, the pathophysiology of epilepsy involves dendritic spine loss and E/I imbalance, which have been reported to exacerbate cortical excitability [42, 43]. Therefore, we propose that the pathologic basis of the increased susceptibility to epilepsy in ASD rats may be related to abnormalities in neuronal and synaptic development, a possibility that will require further investigation in future studies.

### 4.2. Increased Epilepsy Susceptibility in ASD Rats

PTZ induces seizures by causing neuronal hyperexcitability through glutamate excitotoxicity, oxidative stress, and neuroinflammation, particularly dysfunction in the transmission of the inhibitory neurotransmitter γ‐aminobutyric acid (GABA). The characteristics of PTZ‐induced epilepsy in animal models are similar to those in humans, and, therefore, PTZ is often used in the study of seizures [44]. Consistent with prior studies, our results indicated that the rats prenatally exposed to VPA exhibit high sensitivity to PTZ‐induced seizures, characterized by reduced seizure latency and prolonged seizure duration. The VPA‐induced model, originally referred to as the rat autism model, was constructed by Schneider and Przewłocki [28] and reported to exhibit sensory hypersensitivity, repetitive behaviors, and social deficits. Further studies confirmed that the embryonic exposure to VPA enhances local recurrent connectivity and reduces intrinsic excitability in neocortical pyramidal neurons of ASD rat models. These alterations create a hyperconnected yet hypoexcitable state, rendering cortical modules prone to epileptiform activity and sensitive to external stimuli [15]. Considering the altered fetal brain development experimentally induced by sodium valproate in rodent models, it can be hypothesized that changes in neuronal connectivity and excitability or cytoarchitectural changes and/or migratory abnormalities may lead to the formation of epileptogenic cortical regions. Abnormalities in fetal brain development may increase the risk of developing epilepsy. Błaszczyk et al. [45] found that embryonic VPA‐exposed offspring showed disturbed neural activity associated with enhanced neuronal density in the PFC, but not somatosensory area. In the study cohort of Dreier et al. [46], nearly one in 10 children of epileptic mothers who used valproate during pregnancy developed epilepsy, and they concluded that the risk of epilepsy did not vary according to whether the mother used high, medium, or low doses of valproate.

Another study found that rats with VPA‐induced ASD displayed higher susceptibility to electrical ignition‐induced seizures at 4 weeks of age [47], reinforcing that the exposure to VPA during the embryonic stage inhibits brain functions. Based on our morphological findings, we suggest that abnormal neuronal development and PFC morphological disruption induced by prenatal exposure to VPA are the potential pathological basis for the increased susceptibility to PTZ‐induced epilepsy in ASD rats.

Our results reinforce that the prenatal exposure to VPA increases the sensitivity to PTZ‐induced epilepsy.

### 4.3. Relationship Between PFC 5‐HT1A Regulation and Pyramidal Neuron Excitability

Dysregulation of the 5‐HT system has been reported in ASD and has been implicated in abnormal neurodevelopment as well as increased susceptibility to epileptiform activity [48]. Alterations in serotonergic signaling, including changes in 5‐HT1AR function, have been identified in several ASD‐related experimental models and may contribute to abnormalities in cortical circuit regulation. Previous studies have demonstrated that 5‐HT1ARs are widely expressed in cortical regions, including the insular cortex and PFC, where they participate in the modulation of excitatory synaptic transmission [49]. In the present study, ASD rats exhibited significantly reduced 5‐HT1AR expression in the PFC, further supporting the hypothesis of 5‐HT system dysregulation in ASD‐related epilepsy.

The 5‐HT1AR agonist 8‐OH‐DPAT has been reported to exert anticonvulsant effects in several models. Previous studies have shown that 8‐OH‐DPAT reduces seizure susceptibility and attenuates seizure severity in chemically induced seizure models [50]. In particular, Lopez‐Meraz et al. reported that 8‐OH‐DPAT reduced the incidence of tonic seizures and mortality in PTZ‐induced seizures, supporting a potential anticonvulsant role of serotonergic signaling. We speculate that the disruption of E/I balance resulting from the abnormal excitation of excitatory neurons and inhibition of inhibitory neurons increased the susceptibility to epilepsy in ASD rats.

sAP reflects the intrinsic excitability of neurons and the modulatory state of synaptic inputs. In this study, the frequency of sAP issuance from PFC pyramidal neurons was reduced in ASD rats but exhibited a significant increase following the administration of PTZ. Regarding mIPSC and mEPSC, PTZ administration led to a significant increase in the frequency of mEPSC issuance but did not significantly affect the frequency and amplitude of mIPSC in ASD rats. These findings suggest that intrinsic neuronal firing activity is suppressed under baseline conditions in ASD rats, while excitatory synaptic drive becomes enhanced during PTZ challenge. This challenges the findings of a study, which reported that the loss of pyramidal neurons in the hippocampus of rats may be offset by the increase in neuronal activity as a coping mechanism to maintain the network’s function. Such compensatory mechanisms may increase latent neuronal excitability and sensitivity to convulsant stimulation [51]. In a 2‐week‐old rat model of VPA‐induced ASD, the PFC showed hyperplasticity, along with NMDAR overexpression; this suggests that the embryonic exposure to VPA may significantly enhance the ability of neocortical pyramidal neurons to form local recurrent connections and reduce the intrinsic excitability of pyramidal neurons, with localized hyperconnectivity rendering the cortical modules more sensitive to external stimuli [15].

Golgi staining revealed a decrease in the dendritic spine density in the PFC, an alteration that directly reduces the number of functional synapses and may lead to a decrease in the number of excitatory receptors activated by presynaptically released glutamate. Dendritic spines are the principal structural substrates of excitatory synapses, and reductions in spine density are generally associated with decreased synaptic integration capacity and altered neuronal signaling [39]. Reduced dendritic spine density also reduces the number of synchronously activated synaptic inputs, greatly limiting the ability of neurons to integrate excitatory signals from different branches. The reduction in the number of dendritic spines mainly affects excitatory synapses, leading to a relative predominance of inhibitory inputs and a decrease in the overall neuronal excitability. This is consistent with our results in ASD rats, where PFC pyramidal neurons emitted sAP at a reduced frequency. At the network level, such structural alterations may disrupt cortical circuit organization [52], potentially increasing the susceptibility of PFC neurons to PTZ‐induced hyperexcitability, as reflected by the increased frequency of sAPs and mEPSCs under seizure‐provoking conditions.

Furthermore, our results showed that 5‐HT1AR activation in ASD rats resulted in a significant decrease in both sAP and mEPSC frequencies, confirming that 8‐OH‐DPAT can reduce the sensitivity of PFC pyramidal neurons to the PTZ‐induced excitatory effects. It is well established that mEPSC frequency primarily reflects presynaptic neurotransmitter release probability and/or the number of functional excitatory synaptic inputs, whereas mEPSC amplitude mainly reflects postsynaptic receptor responsiveness [53]. In the present study, PTZ increased mEPSC frequency without affecting amplitude, suggesting that PTZ primarily enhances excitatory synaptic drive rather than altering postsynaptic receptor sensitivity. Combined with the reduced expression of 5‐HT1ARs observed in the PFC of ASD rats, the increased mEPSC frequency may reflect weakened serotonergic modulation of excitatory synaptic transmission. Activation of the remaining 5‐HT1ARs by the selective agonist 8‐OH‐DPAT may partially restore this inhibitory modulation, thereby reducing spontaneous excitatory synaptic events without significantly altering postsynaptic response amplitude.

Koyama et al. [54] studied the involvement of the amygdala complex in emotional and behavioral regulation in animals. The study found that a subpopulation of neurons in this region was consistently suppressed by 8‐OH‐DPAT (1 μM). Importantly, 8‐OH‐DPAT inhibited mIPSC frequency without significantly affecting mIPSC amplitude, suggesting that serotonergic modulation may involve presynaptic mechanisms regulating inhibitory synaptic transmission [54]. Previous studies have documented the potential of 8‐OH‐DPAT in reducing or inhibiting the propagation of epileptic activity from the forebrain to the brainstem. In the present study, the application of 8‐OH‐DPAT in the PFC possibly reduced PTZ sensitivity in this region.

### 4.4. Mechanistic Role of Kir3 Channels

Kir3 channels, also referred to as G protein‐coupled inwardly rectifying potassium (GIRK) channels, serve as the primary effector of 5‐HT1AR activation [55]. These channels facilitate slow hyperpolarizing currents [56], which contribute to resting membrane potential, and regulate neuronal firing patterns [57, 58]. Kir3 channels exhibit high expressions in the brain regions associated with learning and memory, such as the hippocampus and PFC, and play a critical role in maintaining cortical network stability [59]. Mice lacking the GIRK2 gene (encoding Kir3.2 channel) exhibit spontaneous seizures and are more prone to pharmacological seizures induced by γ‐aminobutyric acid antagonists. In the present study, 8‐OH‐DPAT treatment induced inwardly rectifying currents in PFC pyramidal neurons, as evidenced by I‐V curve characteristics; however, these currents were blocked after treatment with the Kir3 channel blocker TP‐Q [60]. The reduction in mEPSC frequency, observed in the presence of TP‐Q, was found to increase again in the presence of 8‐OH‐DPAT, suggesting that the inhibitory effect of neuron hyperpolarization was blocked when the Kir3 channel was specifically blocked, which, in turn, increased excitability. This finding confirmed that 5‐HT1AR‐mediated activation of Kir3 channels is the main mechanism for the reduction in neuronal excitability and seizure activity in ASD.

Our findings support the hypothesis that E/I imbalance is a shared pathological mechanism underlying ASD and epilepsy. Reduced 5‐HT1AR expression in the PFC may exacerbate this imbalance by impairing the inhibitory modulation of excitatory circuits, as evidenced by the increased mEPSC frequency in ASD rats. The observed effects of 8‐OH‐DPAT in reducing seizure activity and restoring E/I balance are consistent with the serotonergic modulation in epilepsy reported previously. In addition, we further demonstrated that the effects produced by activation of 5‐HT1ARs are related to Kir3 channels. Overall, our findings highlight the therapeutic potential of targeting 5‐HT1ARs and Kir3 channels for managing epilepsy and related disorders co‐occurring with ASD. However, the present study remains limited in that we only considered a single 5‐HT1AR agonist and did not investigate dose‐dependent effects. Meanwhile, the activity of muscarinic acetylcholine receptors and others will be the next step of research.

However, several limitations of the present study should be acknowledged. First, only a single 5‐HT1AR agonist was investigated, and dose‐dependent effects relationships were not assessed. Second, the potential contribution of other neuromodulatory pathways, such as muscarinic acetylcholine receptor signaling, remains unclear and should be addressed in future studies. Third, the present work focused exclusively on the PFC and therefore did not examine the involvement of other brain regions that may contribute to ASD‐associated epilepsy. Fourth, all electrophysiological recordings were obtained from ex vivo brain slices, which may not fully capture the dynamic properties of in vivo network dynamics or the physiological regulation of excitation‐inhibition balance. Future studies integrating broader circuit‐level investigations with in vivo experimental approaches will be necessary to validate and expand upon these findings.

## 5. Conclusion

Our experimental results underscore the promising potential of 5‐HT1ARs and Kir3 channels as therapeutic targets for managing epilepsy associated with ASD. More comprehensive studies are needed to translate these findings into clinical practice, particularly the application of 5‐HT1AR modulators, in individuals with ASD and comorbid epilepsy. Further investigations into the molecular mechanisms linking serotonergic dysfunction, E/I imbalance, and seizure susceptibility may provide new avenues for therapeutic interventions to manage neurodevelopmental disorders.

## Author Contributions

Study design: Zhuoqi Li and Tao Sun. Implementation of experiments: Yangyang Sun, Xiaofan Ren, Na Ding, and Xianhao Huo. Data collection: Xianhao Huo, Xin Qian, and Ao Li. Data analysis: Xin Qian, Ao Li, Xiaofan Ren, and Na Ding. Manuscript draft: Zhuoqi Li, Yangyang Sun, and Xianhao Huo.

## Funding

This work was supported by the National Natural Science Foundation of China (Grant 82260282), the Natural Science Foundation of Ningxia (Grants 2022AAC03158 and 2023AAC03202), and the Ningxia Medical University scientific research project (Grant XM2020001).

## Disclosure

All authors have read and approved the final manuscript.

## Conflicts of Interest

The authors declare no conflicts of interest.

## Supporting Information

Additional supporting information can be found online in the Supporting Information section.

## Supporting information


**Supporting Information 1** Figure S1: Changes in the protein expression level of 5‐HT1A in the prefrontal cortex of rats in the control group and the Autism group. (uncropped original data).

**Supporting Information 2** Figure S2: Changes in the protein expression level of 5‐HT1A in the prefrontal cortex of rats in the control group, the Autism group, the Autism + Vehicle group, and Autism + 8‐OH‐DPAT group. (uncropped original data).

## Data Availability

The data that support the findings of this study are available from the corresponding author upon reasonable request.
