# Supplementary figures and images for: Prefrontal Cortex 5‐HT1A Receptor‐Coupled Inwardly Rectifying Potassium Channels Decreased Seizure Susceptibility in Rat Models With Autism Spectrum Disorder
Source: Neural Plast. 2026 May 5;2026:4005949. doi: 10.1155/np/4005949 (PMC13140190; doi:10.1155/np/4005949)

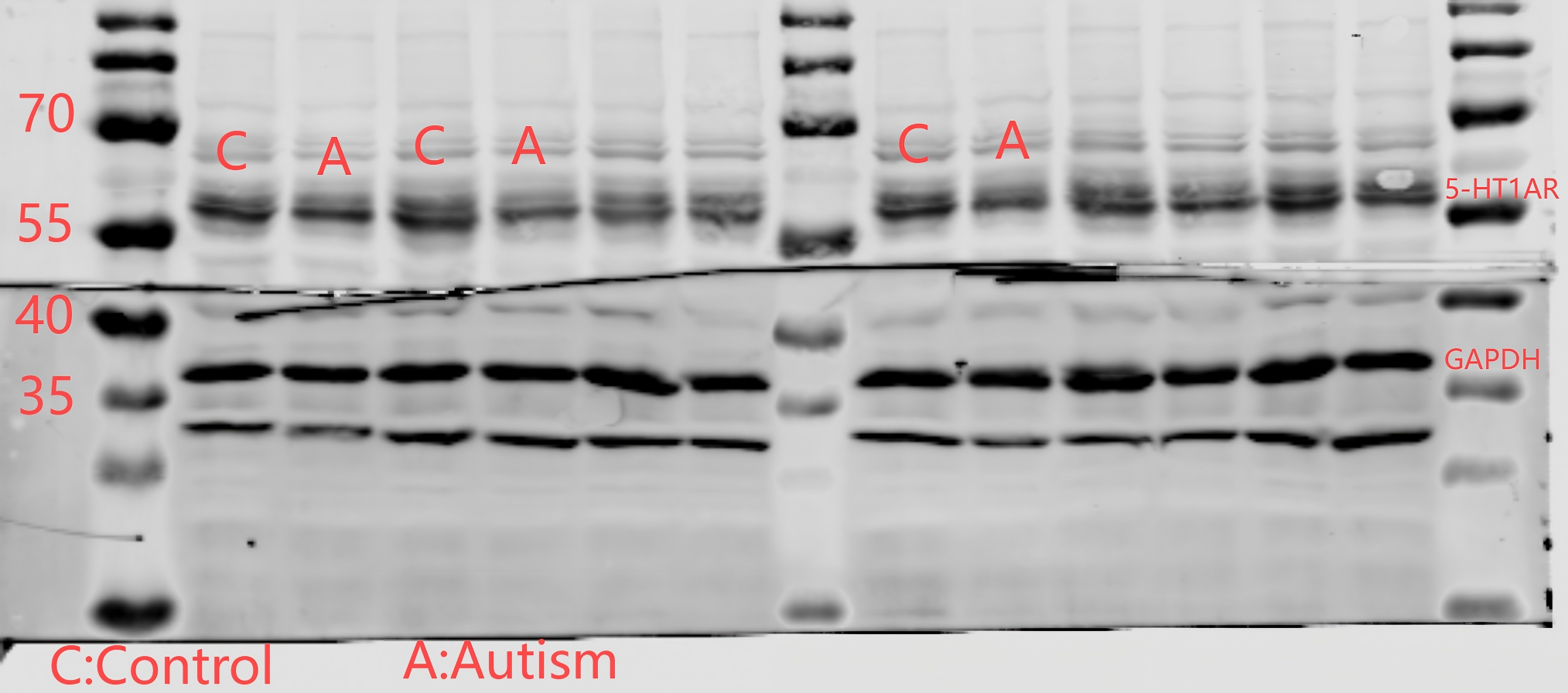

Supplement: Supplementary file 1 — Supporting Information 1 Figure S1: Changes in the protein expression level of 5‐HT1A in the prefrontal cortex of rats in the control group and the Autism group. (uncropped original data). [file NP-2026-4005949-s002.tif]

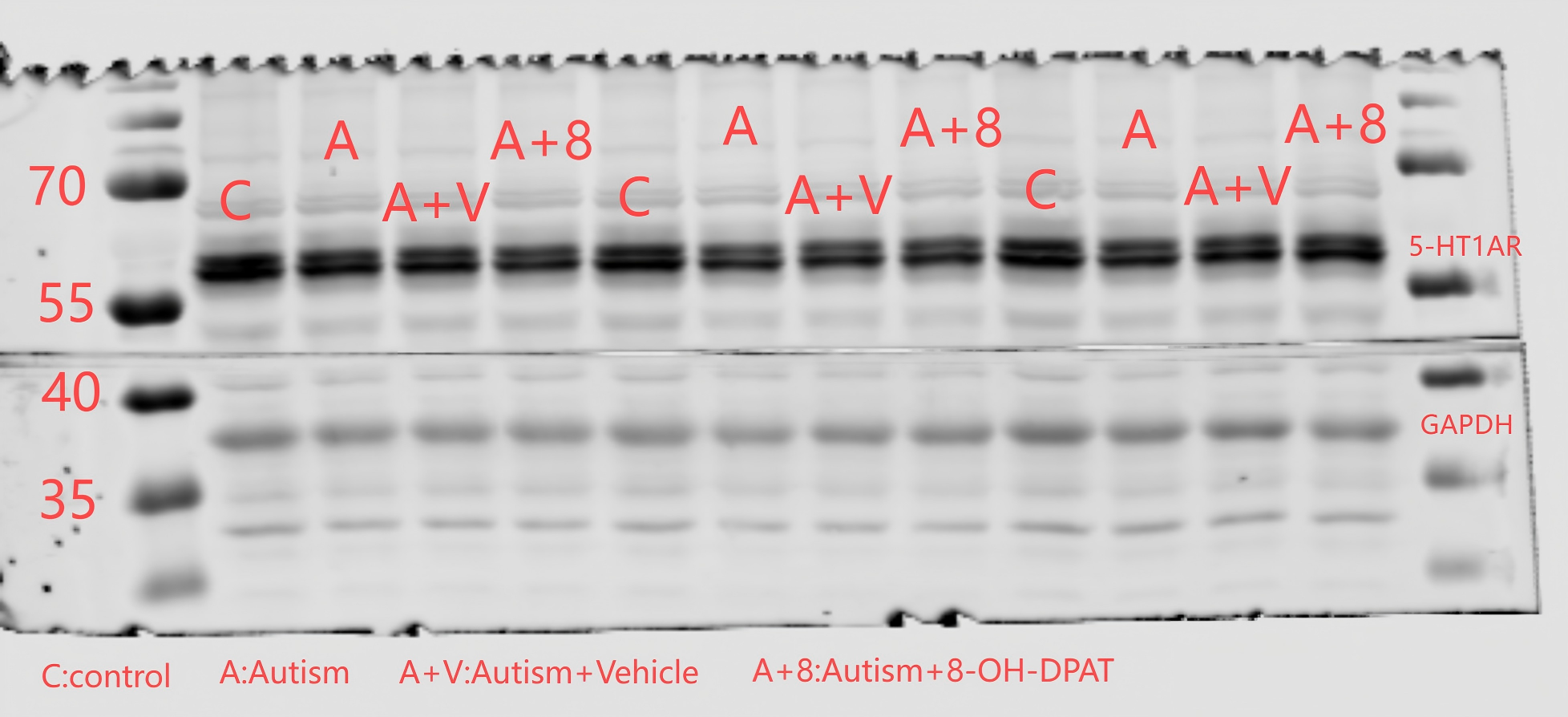

Supplement: Supplementary file 2 — Supporting Information 2 Figure S2: Changes in the protein expression level of 5‐HT1A in the prefrontal cortex of rats in the control group, the Autism group, the Autism + Vehicle group, and Autism + 8‐OH‐DPAT group. (uncropped original data). [file NP-2026-4005949-s001.tif]
